# Validity of the Arabic Version of the PROMIS Anxiety and PROMIS Depression in Cancer Questionnaires: Measuring Depression and Anxiety in Oncologic Patients in Saudi Arabia—A Rasch Analysis Study

**DOI:** 10.3390/jcm14248774

**Published:** 2025-12-11

**Authors:** Hadeel R. Bakhsh, Bodor Bin sheeha, Luigi Tesio, Anna Simone, Stefano Scarano, Nouf Alowain, Ghada A. Bin Dayel, Monira I. Aldhahi, Rehab Alhasani, Antonio Caronni

**Affiliations:** 1 Department of Rehabilitation Sciences, College of Health and Rehabilitation Sciences, Princess Nourah bint Abdulrahman University, P.O. Box 84428, Riyadh 11671, Saudi Arabia; bhbinsheeha@pnu.edu.sa (B.B.s.);; 2 Department of Neurorehabilitation Sciences, IRCCS Istituto Auxologico Italiano, 20122 Milan, Italya.caronni@auxologico.it (A.C.); 3 Department of Biomedical Sciences for Health, University of Milan, 20133 Milan, Italy

**Keywords:** PROMIS, depression, anxiety, Rasch analysis, outcome measures, Arabic, oncology

## Abstract

**Background/Objectives**: The cancer experience has a significant affective impact on patients, often causing anxiety and depression. Given the importance of this condition, there is a true need for psychometrically valid and culturally appropriate assessment tools for anxiety and depression in this condition. This is also true for Arabic-speaking populations. This study evaluates the measurement properties of the PROMIS Depression in Cancer (PROMIS-Ca-D) and Anxiety in Cancer (PROMIS-Ca-A) questionnaires, part of the Patient-Reported Outcomes Measurement Information System^®^ (PROMIS^®^), for assessing depression and anxiety in Saudi Arabian cancer patients. **Methods**: The PROMIS-Ca-D was translated into Arabic and subsequently tested with 30 participants from five Arabic-speaking countries. The PROMIS-Ca-A had been previously translated into Arabic. The second phase recruited 213 cancer patients in Riyadh, Saudi Arabia, who completed the PROMIS-Ca-D and PROMIS-Ca-A. Rasch analysis (rating scale model) was used to assess category functioning, item fit, unidimensionality, differential item functioning, and measures reliability. **Results**: The translation process confirmed the cultural appropriateness of the Arabic PROMIS-Ca-D. In the validation cohort (N = 213), Rasch analysis indicated excellent reliability for both scales. Although disordered modal thresholds and signs of multidimensionality were observed, control analyses confirmed that these features did not compromise the item calibrations or the person’s measures. Item fit was adequate, and Differential Item Functioning was negligible. However, suboptimal item-person targeting was noted. **Conclusions**: The Arabic PROMIS-Ca-D and PROMIS-Ca-A are psychometrically sound instruments for evaluating psychological distress in Arabic-speaking cancer patients. Future research should focus on assessing responsiveness and evaluating metric equivalence with legacy measures.

## 1. Introduction

The global incidence of cancer, the second most significant cause of mortality globally, has been rising, with Global Cancer Statistics reporting 20 million new cancer cases diagnosed in 2022, predicted to increase by 77% in 2050 [1].

Oncology patients often undergo various procedures and treatments, which can be emotionally challenging and provoke anxiety and depression [2]. Receiving a cancer diagnosis is also emotionally challenging, and oncology management becomes more complicated without managing psychological distress [3]. Emotional distress cannot be measured directly, but it can be inferred from a patient’s behaviour [4]. Patient-reported outcome measures (PROMs) are essential for such inference [5]. This also applies in oncology, where PROMs are paramount to evaluating and addressing psychosocial issues experienced by cancer patients during diagnosis and treatment [6].

Dozens of questionnaires exist for the measurement of anxiety and depression, and many of these have been translated into Arabic. Furthermore, many of these questionnaires, although generic (i.e., not developed to measure anxiety and depression in a specific disease or condition), have also been validated in cancer patients.

Common generic questionnaires for measuring anxiety and depression include the Hospital Anxiety and Depression Scale (HADS) [7,8], the Depression Anxiety and Stress Scale (DASS) [9,10], and the Patient Health Questionnaire—9 items (PHQ-9) [11,12,13], all of which are validated in Arabic.

Notably, these instruments were developed precisely to quantify affective symptoms (anxiety or depression) in a general medical and internal medicine context (e.g., HADS and PHQ-9) or (like the DASS) were originally designed to quantify affective difficulties in the general population. This developmental context makes these instruments relevant to the population of interest here, namely, people with oncological disease. These individuals, in fact, can be seen either as patients with an internal medical condition (and thus having an affective reaction to that condition) or as a general population (i.e., primitively without a psychiatric illness) undergoing an enormous stressor.

The questionnaires cited here have been used, albeit in a limited number of studies, in Arabic oncologic patients. For example, the Arabic version of the PHQ-9 was recently used in a sample of women with oncological disease [14], as was the DASS in a similar sample [15]. The HADS has also been used in a sample of Arab people with cancer [16].

However, it must also be noted that studies evaluating the psychometric functioning of the Arabic versions of the HADS and DASS have raised a possible issue of cross-cultural validity. Indeed, it has been reported that key psychometric features of the original DASS and HADS do not replicate well in Arabic-speaking populations [10,17].

Alongside these generic measures of anxiety and depression, there are also disease-specific instruments for the quantification of anxiety and depression in the oncologic patient. Generic and specific scales each have their own strengths and should be understood as complementary to one another, rather than antagonistic.

The main strength of the former (generic scales) is that they allow comparisons between different groups. For example, generic instruments allow for the comparison of the measured variable across different diseases (e.g., depression in oncological disease vs. depression in bereavement vs. depression in neurological disease). The latter (specific scales) can capture critical and perhaps unique aspects of a specific condition. This, hypothetically, improves the scale’s sensitivity (making false negatives less likely—in this case, people who, despite suffering from a certain level of anxiety or depression, score as normal on the questionnaire) and its responsiveness.

Regarding specific questionnaires for the quantification of anxiety and depression in cancer, the Patient-Reported Outcomes Measurement Information System © (PROMIS ©)—an initiative from US-based academic institutions and the National Institutes of Health (NIH) to “establish a national resource for precise and efficient measurement of patient-reported symptoms” [18]—has included an ad hoc measure for this condition among its questionnaires.

PROMIS questionnaires were developed with the aim of improving and, in some respects, overcoming legacy measures. The objective was to measure the same variables as traditional instruments, but to do so in a more efficient, precise, and flexible manner. To achieve these goals, PROMIS measures were not developed using traditional statistical techniques from Classical Test Theory (CTT), as is common for legacy measures, but rather using modern statistical techniques from Item Response Theory (IRT) [18]. A stated goal of PROMIS is to develop measures that allow for comparison “across studies and diseases”.

PROMIS is today a set of person-centred measures used to quantify physical, mental, and social health. Among the measures included in PROMIS are physical health domains, such as fatigue and pain, as well as measures of emotional distress (including anxiety and depression). PROMIS provides both generic measures and measures designed specifically for certain conditions, such as cancer [18,19].

The PROMIS Depression in Cancer (PROMIS-Ca-D) and Anxiety in Cancer (PROMIS-Ca-A) are two item sets designed as specific measures of depression and anxiety in oncological disease. These instruments have been translated into more than ten languages [6,20,21,22]. However, while an Arabic version of the PROMIS-Ca-A was available, the Arabic version of the PROMIS-Ca-D did not exist.

The first aim of this work is to develop an Arabic translation of the PROMIS-Ca-D that is culturally equivalent to the source instrument, i.e., the American version of the PROMIS-Ca-D [18]. Here, “culturally equivalent” means that the variable quantified by the original version of the PROMIS-Ca-D is the same as that captured by the new Arabic translation of the questionnaire. The second aim of the study is to evaluate the measurement properties of the PROMIS-Ca-D and PROMIS-Ca-A questionnaires in Arabic cancer patients. To this end, a robust metrological technique, Rasch analysis, will be used [4,23,24].

This cultural and psychometric validation project of the PROMIS-Ca-A and PROMIS-Ca-D questionnaires is one stage of a more ambitious, ongoing project that has already yielded results. This broader project consists of the translation and cultural validation of multiple PROMIS instruments into Arabic (e.g., [25,26,27]).

## 2. Materials and Methods

This study consisted of two phases, both utilizing a cross-sectional observational design. The preliminary phase (Phase 1) involved translating the PROMIS-Ca-D questionnaire from English to Arabic to obtain a version culturally equivalent to the original instrument. In the second phase (Phase 2), the newly translated PROMIS-Ca-D and the PROMIS-Ca-A (which had already been translated into Arabic in a previous study [26]) were administered to a sample of patients diagnosed with a malignant tumour, and the data were analyzed using Rasch analysis. Details on the two questionnaires assessed in this study, as well as both phases of the current study, are provided in the following sections. The study was conducted in accordance with the Declaration of Helsinki. Institutional Review Board approval was obtained from Princess Nourah bint Abdulrahman University (IRB Log Number: 22-0167), KAAUH (RO-2023-P-019), and KFMC (H-01-R-012).

### 2.1. Outcome Measures

The PROMIS-Ca-A questionnaire comprises 22 items, 20 of which are from the PROMIS Anxiety Bank v1.0 and two are unique to the PROMIS-Cancer Supplement project [19,28], covering anxiety facets such as fear and hyperarousal.

The PROMIS-Ca-D questionnaire comprises 30 items, 23 of which are from the PROMIS Depression Bank v.1.0, and seven are unique to the PROMIS Cancer Supplement [28]. The items assess mood symptoms, including guilt, loss of interest, and fatigue.

Both the PROMIS-Ca-D and PROMIS-Ca-A questionnaires rated symptoms using five categories (1 = Never, 2 = Rarely, 3 = Sometimes, 4 = Often, and 5 = Always). This response structure was identical for all items in both questionnaires. Higher questionnaire total scores indicate higher levels of anxiety or depression [29].

### 2.2. Phase 1: Translation and Cross-Cultural Adaptation of the PROMIS-Ca-D

In April 2022, the authors obtained licences and authorisation from the PROMIS Health Organization to translate the PROMIS-Ca-D item bank into Arabic.

The translation of the PROMIS-Ca-D was conducted according to the Functional Assessment of Chronic Illness Therapy (FACIT) translation protocol [30]. This multi-step process involved two independent forward translations into Arabic, a reconciliation, and a back-translation into English by a native English speaker. The back-translation was compared with the original source text to identify discrepancies. Final consistency checks were performed by the FACIT team in collaboration with the PROMIS Statistical Centre between the questionnaire back translated from Arabic to English and the source English version.

The authors (BHB, HRB, MIA, and RA) conducted cognitive interviews and pilot testing. In this regard, the importance of the cognitive debriefing step must be emphasized, as it ensures that participants (in this case, patients) effectively understand the items of the translated questionnaire in their original intended sense.

Full details are available in the supplements. Supplementary Material S1 details the full translation and cross-cultural adaptation process following the FACIT methodology. Supplementary Material S2 summarizes item-level translation issues, cognitive debriefing feedback, and final wording decisions.

### 2.3. Phase 2: Rasch Analysis of the PROMIS-Ca-D and PROMIS-Ca-A Questionnaires

The Rasch analysis was run on data from a convenience sample of patients diagnosed with malignant tumours who received cancer treatment between April and August 2023 at King Fahad Medical City, Riyadh, Saudi Arabia.

A total of 250 patients were invited to participate in this study. Regarding sample size for the Rasch analysis, 150 participants provide accurate item calibrations and measures for most purposes, with 250 respondents providing robust estimates even in high-stakes tests [31].

The inclusion criteria were age ≥ 18 years, diagnosis of malignant tumour, currently receiving anti-cancer treatment or surgery for cancer treatment in the past 14 days, ability to read and comprehend Arabic, and ability to provide consent. Participants were excluded if they were diagnosed with benign tumours, were in remission, or had a substantial cognitive impairment. Of the 250 participants asked to participate in this study, 15 declined, and 22 patients did not meet the inclusion criteria. In total, 213 participants were included in the final analysis.

To further verify that the sample size recruited here was sufficient to obtain stable item calibrations, statistical simulations were conducted. These simulations, detailed in Section S1.1 of Supplementary Materials S3, confirmed that 213 participants are sufficient.

The participants were approached at the outpatient oncology clinic while waiting for visits. Occupational therapy interns explained the study and obtained consent. Participants completed a questionnaire using Microsoft Forms or paper-and-pencil.

#### Technical Details of the Rasch Analysis Procedure

Rasch analysis [23,24] was used to assess the construct validity and measurement precision of the two questionnaires. The Rating Scale Model (RSM) was applied using Winsteps software (5.6.1) [32]. The RSM was deemed appropriate over more complex models (such as the Partial Credit Model) because all items in both questionnaires shared the identical response category structure (five categories from ‘Never’ to ‘Always’). This uniformity across items is the specific condition that makes the RSM a suitable choice. Furthermore, we preferred the RSM as it is a more parsimonious model.

The following psychometric features were examined:

1. Categories functioning,
2. Data-model fit,
3. Questionnaire’s dimensionality,
4. Questionnaire’s maps,
5. Differential Item Functioning (DIF) and
6. Persons’ measures reliability.

These psychometric features (which are detailed in depth in Supplementary Materials S3) were assessed for the two questionnaires separately, following the order listed above. In this way, they represent successive stages of the analysis, serving as true “diagnostic steps” for evaluating the questionnaire’s functioning.

We stated above that Rasch analysis was used to assess the construct validity of the two questionnaires. In this regard, studying the construct validity of an instrument means testing hypotheses about it using statistical models [33]. Assessing construct validity means evaluating the “*degree [to which] the scores of a measurement instrument are consistent with hypotheses.*” These hypotheses can concern the relationship of the measure with other measures (external validity), but also the internal structure of the questionnaire [33]. These hypotheses about the internal structure largely coincide with the “diagnostic steps” listed above: namely, that participants use the response categories correctly (category functioning), that the questionnaire is unidimensional, and the central hypothesis that the data fit the Rasch model.

Furthermore, using Rasch analysis as a tool for validity assessment is fully aligned with a modern definition of validity (e.g., [34]), which posits that an instrument is a valid measure of a construct if, and only if, (1) the construct exists, and (2) variations in the construct causally produce variations in the measurement outcomes. This approach is central to Rasch analysis, which is built entirely on the assumption that the latent variable (the construct) exists and that the amount of this variable causally determines the probability of responding to items and, consequently, the resulting person measures and item calibrations.

The correct functioning of the categories—i.e., that higher category numerals reflect more of the measured quantity (here, depression and anxiety)—can be assessed by considering the mean category measures and the modal (Andrich’s) thresholds [35,36,37].

Arguably, the most rigorous way to demonstrate correct category functioning is to show that both the mean category measures and the modal thresholds are ordered.

In the case of the PROMIS questionnaires considered here, ordered mean category measures mean that, for a given item, participants who chose (for example) category 2 measure, on average, more than those who chose category 1; and participants who chose category 3 measure, on average, more than those who chose category 2. Demonstrating that participants who gave a higher score also have higher measures (i.e., a greater quantity of the latent variable) effectively defines an ascending monotonic relationship between the category numerals and the measures.

Modal thresholds are values (levels of the latent variable, i.e., measures) at which a person has equal probability of scoring in a given category (e.g., 2) or the immediately successive category (e.g., 3). Demonstrating that the modal thresholds are ordered means showing that the latent variable level required to have an equal probability of scoring 1 or 2 is lower than the level required for scoring 2 or 3, and so on. It is clear that this also defines an ascending monotonic relationship between the thresholds (and thus adjacent categories) and the measured variable.

If the category analysis shows what is called “disordering,” i.e., the presumed monotonic relationship is not complied with, a typical solution is to create a simpler item category structure by collapsing two or more categories into a new, broader category.

However, simulation studies have shown that disordered modal thresholds do not necessarily indicate malfunctioning categories [35], although they do flag a contradiction between the a priori intended progression of the categories and their observed hierarchy (i.e., the disordered category is never the most probable).

With this limitation in mind, the study proceeds with the main analysis as long as the average measures are ordered. However, for completeness, if disordered thresholds are found, a supplementary analysis collapsing the categories to fix the disordered modal thresholds is also provided in Supplementary Materials S3.

Infit and outfit Mean square (MNSQ) and z-standardized (ZSTD) statistics were calculated to investigate whether the items fit the Rasch (RSM) model. An item was considered misfitting if its infit MNSQ was >1.5 and/or outfit MNSQ > 2.0 [38]. Different thresholds were used for infit and outfit MNSQ since outfit MNSQ, sensitive to outliers, can be severely inflated by a few unexpected responses (see Supplementary Materials S3). ZSTD was used to test the null hypothesis of no difference between data and the model’s expectation. More precisely, the MNSQ can be considered an effect size of the distance between data and the model. If this departure is statistically significant, it is tested with the ZSTD. A large departure per the MNSQ value was statistically significant at *p* < 0.05 if the corresponding ZSTD was >1.96.

The Principal Component Analysis (PCA) was run on the model’s standardized residuals to ascertain whether the participants’ responses to the items were unidimensional (i.e., reflecting only the variable grasped by the Rasch model) or if they were affected by a secondary variable. Principal components (PCs) with eigenvalue >2.0 point out hidden variables affecting item scores in addition to the Rasch model variable. If multidimensionality was found, whether this causes harm to measurements is tested as performed elsewhere [39].

It is important to clarify at this point that Rasch analysis is founded on three main assumptions: (i) a monotonic relationship between the item categories and the measured variable (category functioning); (ii) participant responses to items that do not substantially deviate from the model’s predictions (data-model fit); and (iii) unidimensionality. If these assumptions are met, it is possible to extract interval-level measures from the questionnaire’s ordinal scores.

Items, thresholds and person maps show the items and thresholds calibration and the persons’ measures (logits) graphically along the line of the measured construct. Item maps allow for the evaluation of how adequately questionnaire items assess the construct of interest.

Maps also illustrate how items’ difficulties match participants’ ability, i.e., item-persons targeting. Items are “on target” with the sample when the person’s mean ability is within 0.5 logits of the item mean (0 by convention) [32].

Differential Item Functioning (DIF) corrupts an item if the item shows a different difficulty in being endorsed across classes of respondents. DIF was tested for sex (male vs. female), age (<40 years, ≥40 & <60 years, ≥60 years), education level (uneducated, primary or secondary school, high school, university), pre-existing mental disorder (yes vs. no), social status (married vs. alone), cancer localisation (single vs. multiple), time from cancer diagnosis (≤1 year, >1 & ≤5 years, >5 years) and employment (unemployed vs. student or worker vs. retired).

A two-step procedure was also followed in the DIF analysis. DIF was present if: (i) the item calibrations were significantly different in the two groups of respondents (*p* < 0.05, two-sided *t*-test), and (ii) this difference was >0.5 logits [40]. Similarly to multidimensionality, in the eventuality that DIF is found, it is considered dismissible if the consequences of DIF on the questionnaire’s measures are minor [40]. The case of uniform DIF is only considered [41].

Questionnaires’ reliability was reported as Rasch’s person measures reliability [42,43]. From this index, the number of statistically discernible (at *p* < 0.05) “strata” is obtained, i.e., the number of significantly different levels of depression or anxiety a questionnaire can discern at the single-subject level [44].

As reported above, Winsteps (version 5.6.1) was used for the main analysis reported in the main text. R (version 4.3.2) was used for supplementary analyses, to generate figures, and to run the simulations presented in the Supplementary Materials (to assess the adequacy of the sample size considered here). In these simulations, for reasons of computational efficiency, the R package “TAM” (version 4.3.25) was used to fit the RSM [45].

## 3. Results

Thirty healthy adults, six from Saudi Arabia, Egypt, Jordan, Kuwait, and Morocco, participated in the translation and cognitive debriefing process (Table 1). The cognitive debriefing results showed excellent endorsement for comprehension of item instructions and response choices of the PROMIS-Ca-D items. However, two participants considered two items unclear, which required the second level of analysis (Supplementary Materials S2). Item ‘EDANG09—I felt angry’ bore semantic similarities to Item ‘EDANG29—I felt irritable’. To distinguish between irritability and anger, we kept ‘I felt angry’ as the first item and rewrote the second in Arabic. After linguistic validation, the final Arabic translation of the questionnaires is now available at https://www.healthmeasures.net/explore-measurement-systems/promis/intro-to-promis/available-translations (accessed on 30 December 2024).

### Rasch Analysis of the PROMIS-Ca-D and PROMIS-Ca-A Questionnaires

Rasch analysis was run on 213 questionnaires from as many participants (age: mean = 49 years, SD = 15.9 years; 58% females; Table 2).

Categories’ average measures were ordered for both questionnaires even if both questionnaires also demonstrated disordered modal thresholds, with category 2 never being the modal one (Table 3). Because of evidence of ordered mean categories’ measures and the robustness of the Rasch analysis to modal thresholds disordering (see Section 4), the main analysis is carried out with the original items’ categories structure.

Nevertheless, for completeness, an additional analysis is presented in Supplementary Materials S3, in which the original categories 2 and 3 have been collapsed to obtain ordered categories’ mean measures and modal thresholds.

In support of the choice to retain the original category structure for the main analysis, when comparing the item calibration obtained from the questionnaire with the original categories (and thus disordered modal thresholds) to the item calibration obtained with categories 2 and 3 collapsed (and thus ordered thresholds), the calibration difference is minimal (at most < 0.25 logits in absolute value). The difference in infit is also negligible in the two scenarios. Considered overall, these results, which are detailed in Supplementary Materials S3, indicate that the threshold disordering has only a minimal impact on item calibration, i.e., on the de facto tool subsequently used to measure persons.

All 22 items of the PROMIS-Ca-A had a satisfactory fit (Table 4). One item only of the 30 PROMIS-Ca-D items (EDDEP07-“I moved away from others”) was misfitting because of large (MNSQ = 1.61) and statistically significant (ZSTD = 4.84) infit (Table 5).

It is noteworthy that nine items of the PROMIS-Ca-A questionnaire and nine items of the PROMIS-Ca-D questionnaire showed a statistically significant deviation from the model’s expectation (per their ZSTD infit or outfit value). However, with the exception of item EDDEP07 (discussed above), this statistical significance did not represent a practical misfit. For all these other items, the magnitude of the deviation from the model, as quantified by the MNSQ statistic, remained within the acceptable boundaries.

Regarding the dimensionality analysis of the PROMIS-Ca-A, the PCA showed that this questionnaire is three-dimensional, with the first and second PC having eigenvalues of 2.46 and 2.24, respectively. A control analysis assessing the multidimensionality impact on the PROMIS-Ca-A measures showed that the hidden variables discovered with the PCA have no relevant harm on respondents’ measures (Supplementary Materials S3).

The PCA of the PROMIS-Ca-D residuals highlighted three PCs with eigenvalue > 2.0 (2.67, 2.13, and 2.05, respectively), indicating three unwanted variables driving the items’ scores. Similarly to the PROMIS-Ca-A, this multidimensionality is likely to cause no harm to the measures (Supplementary Materials S3).

**Figure 1:**
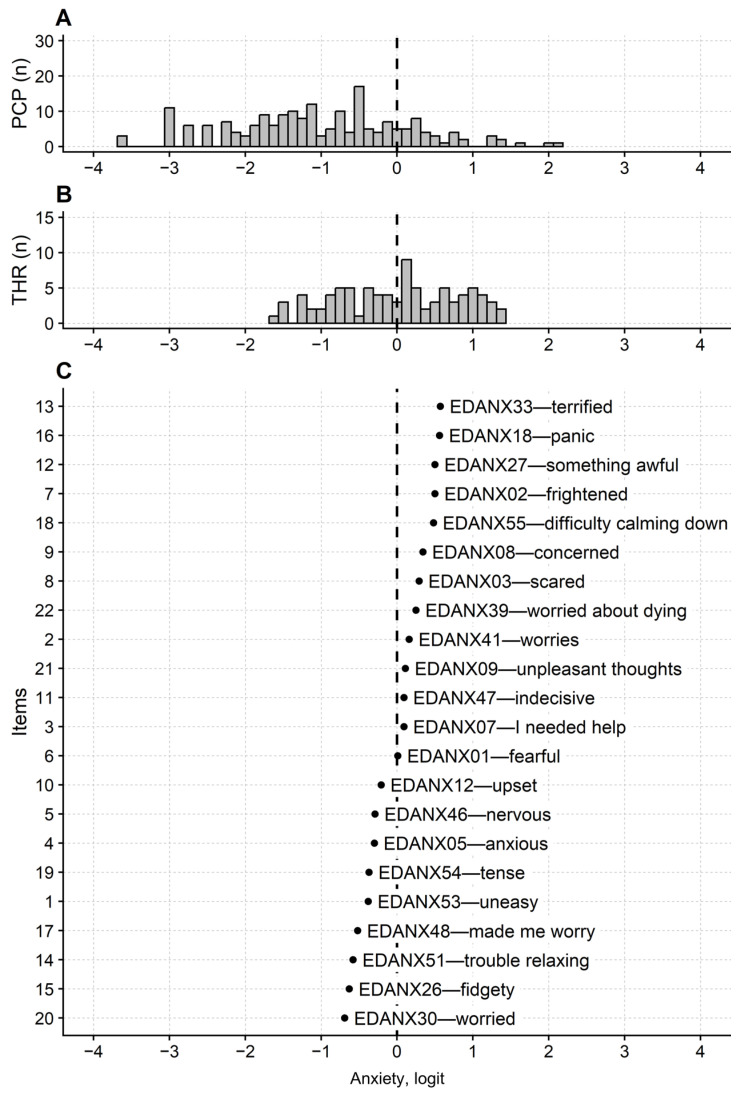
Questionnaire’s maps of the PROMIS Anxiety in Cancer questionnaire. The persons (**A**), thresholds (**B**) and items (**C**) maps are displayed. THR: thresholds; PCP: participants. n: number of. X-axes: latent variable, i.e., anxiety severity, on an interval scale with logit as the measurement unit. The distribution frequency of the Andrich (modal) thresholds is provided regarding the thresholds map. In the items map, items are sorted according to their mean calibration. Each item is labelled with its PROMIS item bank code (EDANX prefix) and a keyword summarizing the item content. Numerals on the y-axis in C give the item number in the PROMIS item bank (1, first item in the item bank and so on). Vertical dashed line marks 0 logit, i.e., the items mean calibration. Note from the distribution frequency in A that most persons (non-extreme persons only are reported) measure less than 0 logits. Moreover, there are no items nor thresholds for precisely measuring persons with less than −2 logit of anxiety. This finding flags a construct validity flaw of the questionnaire when used to measure persons suffering from low, but nonzero, anxiety levels.

**Figure 2:**
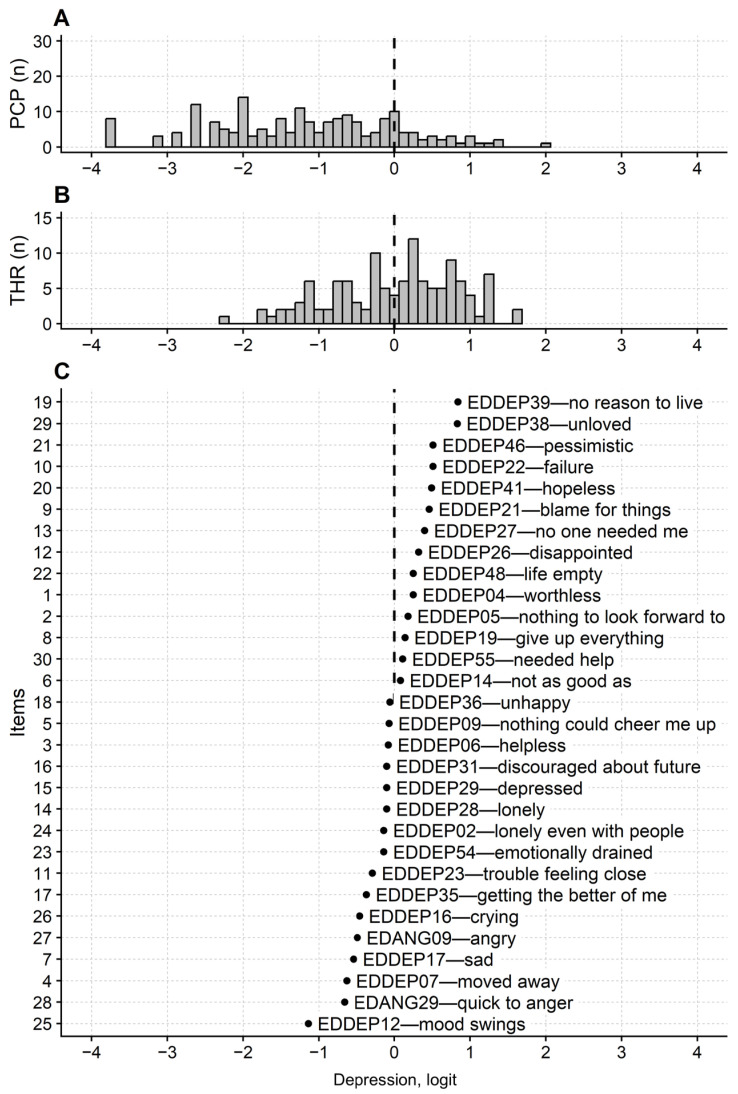
Questionnaire’s maps of the PROMIS Depression in Cancer questionnaire. Persons (**A**), thresholds (**B**) and items (**C**) maps. X-axes: latent variable, i.e., depression severity, on an interval scale with logit as the measurement unit. Abbreviations and other details as in Figure 1. Note also, in this case, the lack of items and thresholds for precise measurement of persons with low, nonzero depression levels (less than −2.5 logit).

Items, thresholds, and person maps of the PROMIS-Ca-A and PROMIS-Ca-D are provided in Figure 1 and Figure 2, respectively. In terms of the items’ calibration, the item map of the PROMIS-Ca-A spanned 1.26 logit from item “EDANX30—I felt worried” (−0.69 logit), to item “EDANX33—I felt terrified” (0.57 logit). When the items’ thresholds are considered alongside the item’s calibrations, the map range is more than doubled, reaching about 3.0 logits (−1.61 to 1.36). However, the person’s map shows that the sample-items targeting is poor, with the persons’ mean measure being −1.02 logit (non-extreme persons only). Figure 1 also shows that no thresholds are present for measuring the participants with a measure in the range −4 to −2 logit, indicating that the PROMIS-Ca-A could perform suboptimal in measuring persons with low anxiety levels. Finally, 8.0% of the respondents totalled the minimum score, flagging some floor effect of the questionnaire.

Findings about the PROMIS-Ca-D maps are comparable to those reported for the anxiety questionnaire (Figure 2). The item’s map was about 2.0 logits long, from −1.14 (item “EDDEP12—I had mood swings”) to 0.84 logit (“EDDEP39—I felt I had no reason to live”), and when the thresholds are considered, the range increases to 3.84 logit. Poor persons-items targeting was also found with the persons’ mean measure (non-extreme only) being −1.16. In this case, too, no thresholds are found in the range of −4 to −2 logits. Regarding the floor effect, the minimum score was reached by 13.1% of the respondents.

For PROMIS-Ca-A, no DIF was found for gender, education level, mental disorder, social status, single vs. multiple cancer localisations, time from cancer diagnosis, and employment status. Item EDANX18 had a trascurable DIF for age (DIF contrast: −0.78 logit; *p* = 0.008; Supplementary Materials S3). Regarding the PROMIS-Ca-D, no DIF was found for age, the presence of a mental disorder, single vs. multiple localisations and social status. DIF was found for education level, employment status, gender and the time from diagnosis. The most problematic DIF variable was the “employment status”, which caused four items “to DIF”. The largest DIF was for item EDDEP21 (unemployed vs. retired; DIF contrast = 1.67 logit; *p* = 0.005) followed by item EDDEP19 (unemployed vs. retired; DIF contrast = 1.17; *p* = 0.006).

The complete results of the DIF analysis are reported in Supplementary Materials S3. Similarly to multidimensionality, although present, DIF is unlikely to harm the measures from the PROMIS-Ca-A and PROMIS-Ca-D questionnaires.

PROMIS-Ca-A and PROMIS-Ca-D showed excellent person reliability (0.90 and 0.91; non-extreme measures only) with person separation of 2.93 and 3.20, respectively, a value allowing to distinguish four anxiety and depression strata at the single subject level (e.g., mild, moderate, severe, and extreme). Reliability remained fair (0.84 for and 0.81 for the PROMIS-Ca-A and PROMIS-Ca-D, respectively) after including non-extreme and extreme persons’ measures.

The score-to-measure conversions for the two questionnaires are provided in Supplementary Materials S3.

## 4. Discussion

The study translated the PROMIS-Ca-D item bank into Arabic and assessed the construct validity of the PROMIS-Ca-D and PROMIS-Ca-A questionnaires, using Rasch analysis, among cancer patients in Saudi Arabia.

Regarding the translation phase, two items required additional refinement during cognitive debriefing (Supplementary Materials S2). Based on participant comments, the distinction between anger and irritability in Items EDANG09, ‘I felt angry’, and EDANG29, ‘I felt quick to anger’, was unclear because these terms sounded similar in Arabic. After consultation with a professional translator, we retained the first item and modified EDAANG 29 to clearly state it pertains to anger and irritability (Supplementary Materials S2).

Moving to the study’s second phase, both questionnaires were evaluated using RA. The respondents’ measures advanced on average with the item categories of the Arabic version of the PROMIS-Ca-A and PROMIS-Ca-D questionnaires, suggesting a monotonic relationship between the categories’ numerals and measured quantity. However, ordered categories measures were found with disordered modal (Andrich’s) thresholds for both questionnaires.

According to some authors (e.g., Ref. [46]), disordered thresholds point toward serious category malfunctioning, to the point that the item category structure must be modified to obtain a questionnaire with ordered categories and thresholds. Usually, when a questionnaire presents disordered thresholds, two or more categories are collapsed into a new category, with the objective that, upon re-analysis, the questionnaire with the new, artificial category structure will have ordered thresholds. However, not all authors agree with this solution [47]. It is for this reason that this procedure (the merging of adjacent categories into a new category) is presented here as a supplementary analysis.

Disordered thresholds can occur for different reasons and not necessarily because the categories themselves are disordered. For example, disordered thresholds can occur when an adequately ordered category is rarely chosen (i.e., “underused”) [48]. Conversely, ordered thresholds can be found despite serious miscoding of item categories [35].

From a strictly computational standpoint, it is not necessary to impose ordered thresholds to estimate Rasch calibrations [49] (but see [50]). Given these findings, the original category structure can be retained despite disordered modal thresholds, as long as the categories’ average measures are ordered.

Recent guidelines [51,52] also suggest, but do not impose, specific actions to take when disordered thresholds are found. Specifically, the cited guidelines indicate that researchers must decide on a case-by-case basis “to what extent these [disordered thresholds] are disrupting person measurement” [52]. When disordered thresholds are found, it is more reasonable to consider the severity of this disorder rather than automatically applying corrective solutions, such as collapsing categories, which might only improve the statistical aspects of the questionnaire. It is also further specified that collapsing categories with disordered thresholds should be carried out “when appropriate” [51].

However, as mentioned above, for completeness, an additional analysis collapsing the categories is also presented (Supplementary Materials S3). In that analysis, merging the original categories 2 and 3 into a new category 2 allowed both PROMIS-Ca questionnaires to achieve ordered categories’ average measures and modal thresholds.

Regarding this supplementary analysis, it is also worth pointing out that collapsing categories in the event of disordered thresholds should be understood, at most, as a hypothesis to be confirmed experimentally. In this sense, combining adjacent categories after the data are collected (post hoc) does not guarantee finding the same result that would be found if the categories had been combined a priori (i.e., by modifying the instrument) and new data were then collected using that new structure.

Data-model fit results revealed a misfitting item in the PROMIS-Ca-D questionnaire (EDDEP07, “I Moved away from others”), while no misfitting items were found in PROMIS-Ca-A. Given the study’s aim (providing a cross-cultural equivalent version of the PROMIS-Ca-A and PRO-MIS-Ca-D questionnaires), what matters is checking whether there are any issues with the translation. However, supported by the cognitive de-briefing results, we would exclude that this misfit flags a problem with the cross-cultural validation process (Supplementary Materials S2).

Regarding item misfits, it should also be noted that the PROMIS was not primarily developed in the Rasch analysis framework, but different item response theory models were used. In particular, models with multiple discriminating parameters were applied, likely improving the data model fit. Suppose the item’s slope was originally allowed to vary. In that case, it is unsurprising that some misfit is found when the PROMIS questionnaires are assessed in the Rasch analysis, in which, on the contrary, the item’s slope is fixed.

In Rasch analysis, items that do not fit the model are often removed to achieve a 100% “Rasch consistent” questionnaire. While this is a rigorous choice, this solution is not without side effects. Furthermore, given the aim of the current study, it is likely not the most reasonable choice.

Crucially, as the intention of this study was to validate the Arabic version of the PROMIS-Ca-D rather than to develop a new scale, we prioritized fidelity to the original instrument and chose to retain this item, while still reporting its statistical malfunction.

Another universally valid reason for not removing items, especially from an instrument already in use, is content validity. In this step of the Rasch analysis, it is as if two forms of validity are in balance: statistical validity (which prioritizes fit) and content validity (which considers the item’s content, what it addresses, and which aspect of the variable it investigates). A truly valid questionnaire is one that balances these two aspects.

In this regard, the item ‘EDDEP07—“I moved away from others”’ represents social withdrawal, a core and clinically highly relevant symptom of depression. We, therefore, prefer to retain an item that is “statistically noisy” (misfitting) but “clinically essential,” rather than having a scale that is psychometrically “clean” but clinically incomplete.

Finally, while the item’s misfit is certainly beyond the tolerance threshold we set, it must also be said that this infit MNSQ value (1.61) is not catastrophic and is only slightly above the 1.50 threshold we had set. As also reported in Supplementary Materials S3, items with MNSQ between 1.5 and 2.0 are considered unproductive but do not distort measurements [38]. In summary, the most reasonable and cautious choice at this stage is to retain this single misfitting item because the misfit does not appear to reflect a cross-cultural validation problem (the goal of the present study), the misfit is nonetheless small (and likely considered non-substantial by other authors), and the question expressed by the item is relevant to the construct being measured.

Looking at unidimensionality, both questionnaires demonstrated the presence of hidden variables, two for PROMIS-Ca-A and three for PRO-MIS-Ca-D, in addition to the Rasch variable (i.e., anxiety and depression, respectively). However, an extensive set of control analyses detailed in Supplementary Materials S3 was run to assess to what extent multidimensionality harms anxiety and depression measurement. What is shown is that, in practical metrological terms, the additional hidden dimensions do not cause any severe measurement artefact.

The questionnaires’ maps highlight the poor targeting of items, likely the major flaw of the PROMIS-Ca-A and PROMIS-Ca-D questionnaires. After excluding persons who achieved the minimum score, i.e., considering only the respondents suffering some amount of depression or anxiety, the persons’ mean measure was about −1 logit, i.e., one logit below the item mean measure. This finding indicates that the items are too difficult to endorse for the current sample of cancer patients: the problems addressed by the items are too severe for the average anxiety and depression of the sample. Moreover, looking at the maps, it is also apparent that no thresholds and items (i.e., no ticks in the ruler) are available to measure patients suffering very low levels of anxiety or depression.

This issue, however, is not entirely unexpected. Indeed, it has been shown that both PROMIS anxiety and depression questionnaires have the highest precision when administered to patients with moderate to severe symptoms [53,54].

Regarding the DIF analysis, the Arabic version of the PROMIS-Ca-A showed no bias for several important variables, including gender and having single or multiple cancer localisations. This makes PROMIS-Ca-A suitable for comparing the anxiety levels in male and female cancer patients, patients with single cancer localisations, and patients with multiple localisations. In addition, no DIF was found for the time from cancer diagnosis, thus allowing for tracking anxiety from early after diagnosis to the cancer chronic phase.

DIF was found for one item because of age, however, given that the overall DIF size was small and the robustness of the questionnaire total score to even a considerable amount of DIF of its items [55], it is unlikely that the DIF caused by age substantially biased the measures from PROMIS-Ca-A.

DIF was more frequent for the Arabic PROMIS-Ca-D items, with a substantial DIF of four item contrasts caused by employment status. EDDEP21 and EDDEP19 were more difficult to endorse among unemployed than retired patients (DIF contrast up to 1.67 logits). A control analysis was run to understand the measurement artefact caused at the total score level by this DIF, verifying that, also in this case, the malfunctioning of individual items due to DIF did not harm the questionnaire’s measures.

The PROMIS-Ca-A measures are sufficiently free from DIF, indicating they could be distributed to participants of different subgroups without fear of items measuring different constructs across subgroups. A similar conclusion was reached for the Brazilian translation of the PROMIS Anxiety and Depression questionnaires [56].

### Strengths, Limitations and Future Research Directions

Receiving a cancer diagnosis, navigating the diagnostic and therapeutic process, and hearing the prognosis all have clear affective repercussions for patients. These affective repercussions must be monitored and, where appropriate, may also require an intervention, which in turn requires monitoring of its effectiveness. A PROM is the essential, and indeed the only, instrument capable of quantifying this emotional impact. The importance of having optimal instruments to measure this problem is therefore self-evident. In this view, the greatest strength of the work presented here lies precisely in its clinical impact: providing valid measures, here in the Arabic culture, for the quantification of the emotional impact of cancer.

Another true strength of our work lies in the process followed for the translation and cultural adaptation of the original PROMIS-Ca-A and PROMIS-Ca-D items, and in having studied their metrological validity with a rigorous technique rooted in measurement theory, namely Rasch analysis.

The implications of this work extend beyond everyday clinical practice to research. In research, it is worth mentioning the extraordinary importance of having questionnaires that measure the same variable in the same way across different cultures (i.e., international measures of disability, of walking and, as performed here, of anxiety and depression). This allows for the comparison of treatment efficacy in diverse nations and cultures (e.g., ‘Why did a treatment prove effective in Europe but not in Arabia? Is it a problem with the treatment or the measurement tool?’). It also makes it possible to conduct international multicentre studies with the same primary outcome and to aggregate results from different studies in meta-analyses.

Among the limitations was that the study used a convenience sample from a single oncology centre and a single Arabic-speaking country.

Furthermore, we acknowledge that from an oncological perspective, these patients could be profiled in greater detail. For example, by adding information on the type of oncological disease, prognosis, and treatments received—all information that may impact how patients respond to questions about anxiety and depression. For instance, one might speculate that a patient undergoing chemotherapy that causes severe nausea and vomiting might be more prone to endorse “I had difficulty calming down” (item EDANX55 of PROMIS-Ca-A). This would not necessarily be because their anxiety levels are high, but rather as a physiological stress reaction to the chemotherapy-induced nausea. Collecting this information would allow for further investigation of the items’ invariance. Demonstrating that the questionnaire items do not exhibit DIF for these variables would allow us to conclude that the two PROMIS instruments permit the comparison of anxiety and depression severity in patients with different oncological diagnoses, different prognoses, and undergoing different cancer treatments.

It must be stressed that the PROMIS-Ca-A and PROMIS-Ca-D item banks were developed with Item Response Theory, while Rasch analysis was used for this validity study. One could consider it unfair to test a questionnaire “built to run” in one measurement theory within a different framework. However, while scholars embracing Rasch’s analysis consider it distinct from Item Response Theory [57], mathematically, the models of the Rasch family can be viewed as a particular type of Item Response Theory model. Moreover, this study shows that the two PROMIS questionnaires performed well even when assessed using the RA. Confirming its good measurement features in a different theoretical framework should be considered additional evidence of their proper functioning.

Another issue to address is that PROMIS-Ca-A and PROMIS-Ca-D, which we defined as “questionnaires,” originated as item banks. In this regard, it is important to emphasize that the PROMIS initiative itself allows the selection of items from the banks to create instruments for measuring persons. Based on this, and given the results of this study, a future development of this work could be to develop and adapt shortened versions of the PROMIS-Ca-A and PROMIS-Ca-D questionnaires by removing items showing malfunctioning (e.g., the misfitting item).

Finally, it is important to reflect on the cross-cultural equivalence between the Arabic version of PROMIS-Ca-D developed and presented here and the source version. In this study, equivalence between the two PROMIS-Ca-D translations is asserted in light of the rigorous translation and equivalence verification process. In this regard, it is important to emphasize that the FACIT methodology was followed and that the PROMIS organization itself, the developer of the source questionnaire, performed the final consistency checks between the different versions of the questionnaire. It is this process that ensures the Arabic version of PROMIS-Ca-D is equivalent to the original.

However, this does not obviate the need to evaluate in future studies whether the two questionnaires are metrically equivalent (i.e., the same item parameter corresponds to the same quantity of the latent variable in either language). Rasch analysis, which facilitates the study of DIF, is again the optimal tool to demonstrate the metric equivalence of different questionnaire translations (e.g., [58]). This would involve collecting data using both the Arabic and the original language versions; evidence of DIF, indicating differences in item calibrations, would open the possibility that the different item translations are assessing different areas of the same variable, or even different variables altogether. This remains a future objective for this line of research.

This study was aimed at the cultural and psychometric validation of two disease-specific questionnaires for the quantification of anxiety and depression in cancer. As detailed in the introduction, previous studies have measured these two variables in this condition using generic instruments such as the HADS and the DASS. We decided to validate the PROMIS instruments because we had a focus on anxiety and depression in cancer, and because PROMIS offers tools specifically dedicated to this. Furthermore, previous studies had already highlighted some problems with the Arabic versions of generic questionnaires like HADS and DASS. A direction for future research, therefore, is to further evaluate the psychometric functioning of the Arabic versions of these generic instruments, perhaps in this case using Rasch analysis as well. This is also because, as reported in the introduction, generic and disease-specific questionnaires are not antagonistic but rather complement one another.

Along these lines, it is worth noting that such a study (evaluating the Arabic versions of generic instruments) would also provide the opportunity to assess other aspects of the PROMIS questionnaires’ construct validity. For instance, by collecting data with the PROMIS, HADS, and DASS questionnaires from the same sample of individuals, it would be possible to study convergent validity [33]. Of course, for a true construct validity study, this analysis must be preceded by a priori hypotheses regarding the direction and strength of this relationship.

## 5. Conclusions

While the Rasch analysis revealed some flaws, the PROMIS-Ca-A and the PROMIS-Ca-D show satisfactory psychometric features overall, suggesting that these measures are suitable for evaluating psychological distress in cancer patients, including Arabic-speaking patients.

## Figures and Tables

**Table 1:** Demographic characteristics of translation and cognitive debriefing participants (n = 30).

Variables	Total (N = 30)
Age, years, Mean (SD)	35.2 (10.5)
Sex, N of females (%)	15 (50%)
Educational level, N (%)	
High School	11 (37%)
Diploma	7 (23%)
Undergraduate	12 (40%)

**Table 2:** Demographic characteristics of the participants of the Rasch analysis study (n = 213).

Variables	Total (N = 213)
Age, years, Mean (SD)	49 (15.6)
Sex, N of females (%)	123 (58%)
BMI, kg/m^2^, Mean (SD)	25.7 (5.3)
Diagnosis, N (%)	
Breast	37 (17.4)
Colon	32 (15.0)
Leukemia	22 (10.3)
Liver	17 (8.0)
Lymphoma	12 (5.6)
Not specified	20 (9.4)
Time from diagnosis, years, Mean (SD)	2.35 (2.61)
Educational level, N (%)	
Uneducated	21 (10%)
Primary or secondary school	56 (26%)
High School	72 (34%)
University	64 (30%)
Employment status, N (%)	
Student or worker	83 (39%)
Retired	29 (14%)
Unemployed	101 (47%)
Marital status, N (%)	
Married	135 (63%)
Alone (single, divorced or widowed)	78 (37%)

Regarding cancer diagnosis, the sample was heterogeneous; only the five most frequent diagnoses are reported.

**Table 3:** Categories functioning of the PROMIS-Ca-A and PROMIS-Ca-D questionnaires.

Category Label	Observed Average	Modal Thresholds	Infit MnSq	Outfit MnSq
PROMIS-Ca-A				
1 Never	−1.88	None	0.92	0.99
2 Rarely	−0.91	−0.39	1.01	0.79
3 Sometimes	−0.37	−0.92 (*)	0.95	0.97
4 Often	0.09	0.52	1.10	1.22
5 Always	0.71	0.79	1.23	1.37
PROMIS-Ca-D				
1 Never	−2.00	None	0.90	0.99
2 Rarely	−0.83	−0.11	1.17	0.78
3 Sometimes	−0.44	−1.06 (*)	0.99	0.94
4 Often	0.11	0.36	1.05	1.15
5 Always	0.63	0.80	1.21	1.33

Observed average: mean measure (in logits and referred to the item’s difficulty) of the participants scoring in each of the five categories; if categories are ordered, this average measure is expected to increase with the category’s numeral; (*). Modal thresholds: Andrich thresholds (tau parameters) expressed in logits; * marks disordered thresholds; MnSq: mean square. PROMIS-Ca-A and PROMIS-Ca-D questionnaires had disordered modal thresholds, with the threshold between categories 2 and 3 on the right of the threshold between categories 1 and 2. Despite this, the categories’ mean measure was ordered.

**Table 4:** PROMIS-Ca-A Item Calibration.

Item Number	Item Description	Calibration (SE)	Infit MnSq (Zstd)	Outfit MnSq (Zstd)
(13) EDANX33	I felt terrified	0.57 (0.09)	0.85 (−1.25)	0.82 (−0.87)
(16) EDANX18	I had sudden feelings of panic	0.56 (0.09)	0.95 (−0.35)	0.73 (−1.40)
(12) EDANX27	I felt something awful would happen	0.50 (0.09)	0.81 (−1.68)	0.82 (−0.89)
(7) EDANX02	I felt frightened	0.50 (0.09)	0.96 (−0.32)	0.82 (−0.93)
(18) EDANX55	I had difficulty calming down	0.48 (0.09)	1.01 (0.08)	0.80 (−1.05)
(9) EDANX08	I was concerned about my mental health	0.34 (0.9)	1.25 (2.04)	1.27 (1.46)
(8) EDANX03	It scared me when I felt nervous	0.29 (0.09)	1.04 (0.42)	0.90 (−0.53)
(22) EDANX39	I worried about dying	0.25 (0.09)	1.27 (2.29)	1.73 (3.53)
(2) EDANX41	My worries overwhelmed me	0.16 (0.08)	0.89 (−1.05)	1.05 (0.38)
(21) EDANX09	I had unpleasant thoughts that wouldn’t leave my mind	0.11 (0.08)	0.94 (−0.52)	0.84 (−1.00)
(3) EDANX07	I felt like I needed help for my anxiety	0.90 (0.8)	1.10 (0.93)	1.08 (0.53)
(11) EDANX47	I felt indecisive	0.90 (0.08)	0.82 (−1.71)	0.73 (−1.81)
(6) EDANX01	I felt fearful	0.10 (0.08)	0.80 (−1.98)	0.69 (−2.17)
(10) EDANX12	I felt upset	−0.21(0.8)	0.81 (−1.94)	0.73 (−2.06)
(5) EDANX46	I felt nervous	−0.29 (0.8)	1.15 (1.45)	1.24 (1.68)
(4) EDANX05	I felt anxious	−0.30 (0.8)	0.87 (−1.35)	0.94 (−0.42)
(19) EDANX54	I felt tense	−0.37 (0.8)	0.73 (−2.92)	0.82 (−1.37)
(1) EDANX53	I felt uneasy	−0.38 (0.8)	1.30 (2.76)	1.52 (3.45)
(17) EDANX48	Many situations made me worry	−0.52 (0.8)	1.18 (1.77)	1.31 (2.30)
(14) EDANX51	I had trouble relaxing	−0.58 (0.8)	1.41 (3.71	1.46 (3.27)
(15) EDANX26	I felt fidgety	−0.63 (0.8)	1.26 (2.48)	1.46 (3.36)
(20) EDANX30	I felt worried	−0.69 (0.8)	1.04 (0.42)	1.02 (0.17)

Calibration: item calibration, i.e., item difficulty; items with larger calibrations are more difficult to endorse, thus flagging higher anxiety levels. The items are ordered from top to bottom, from the most difficult to the easiest. SE: standard error; MnSq: Mean square; Zstd: Z-standardized.

**Table 5:** PROMIS-Ca-D Item Calibration.

Item Number	Item Description	Calibration (SE)	Infit MnSq (Zstd)	Outfit MnSq (Zstd)
(19) EDDEP39	I felt I had no reason to live	0.84 (0.10)	0.86 (−1.00)	0.70 (−1.03)
(29) EDDEP38	I felt unloved	0.83 (0.10)	1.07 (0.56)	0.83 (−0.51)
(21) EDDEP46	I felt pessimistic	0.51 (0.09)	0.83 (−1.37)	0.57 (−1.92)
(10) EDDEP22	I felt like a failure	0.51 (0.09)	0.82 (−1.48)	0.68 (−1.35)
(20) EDDEP41	I felt hopeless	0.49 (0.09)	0.62 (−3.42)	0.45 (−2.71)
(9) EDDEP21	I felt that I was to blame for things	0.46 (0.09)	1.30 (2.26)	1.31 (1.21)
(13) EDDEP27	I felt that no one needed me	0.40 (0.09)	1.17 (1.33)	0.89 (−0.40)
(12) EDDEP26	I felt disappointed in myself	0.32 (0.09)	0.79 (−1.79)	0.61 (−1.92)
(1) EDDEP04	I felt worthless	0.25 (0.09)	0.78 (−1.98)	0.90 (−0.40)
(22) EDDEP48	I felt my life was empty	0.25 (0.09)	0.79 (−1.80)	0.72 (−1.32)
(2) EDDEP05	I felt like I had nothing to look forward to	0.18 (0.09)	1.16 (1.33)	1.36 (1.58)
(8) EDDEP19	I felt like I wanted to give up everything	0.14 (0.09)	1.24 (1.91)	1.43 (1.85)
(30) EDDEP55	I felt like I needed help for my depression	0.11 (0.09)	1.33 (2.61)	1.12 (0.60)
(6) EDDEP14	I felt that I was not as good as other people	0.08 (0.09)	0.89 (−0.91)	0.84 (−0.77)
(18) EDDEP36	I felt unhappy	−0.06 (0.08)	0.80 (−1.82)	0.77 (−1.21)
(5) EDDEP09	I felt that nothing could cheer me up	−0.07 (0.08)	0.82 (−1.66)	0.70 (−1.68)
(3) EDDEP06	I felt helpless	−0.08 (0.08)	0.86 (−1.31)	0.87 (−0.66)
(15) EDDEP29	I felt depressed	−0.10 (0.08)	0.89 (−0.94)	0.93 (−0.34)
(16) EDDEP31	I felt discouraged about the future	−0.10 (0.08)	0.83 (−1.52)	1.10 (0.56)
(14) EDDEP28	I felt lonely	−0.10 (0.08)	1.05 (0.46)	1.16 (0.88)
(24) EDDEP02	I felt lonely even when I was with other people	−0.14 (0.08)	0.68 (−3.22)	0.57 (−2.72)
(23) EDDEP54	I felt emotionally drained	−0.14 (0.08)	1.09 (0.80)	1.08 (0.46)
(11) EDDEP23	I had trouble feeling close to people	−0.29 (0.08)	1.16 (1.41)	1.15 (0.89)
(17) EDDEP35	I felt that the circumstances of my life were getting the better of me	−0.37 (0.08)	0.85 (−1.41)	0.90 (−0.60)
(26) EDDEP16	I felt like crying	−0.46 (0.08)	1.40 (3.32)	1.75 (3.92)
(27) EDANG09	I felt angry	−0.49 (0.08)	1.20 (1.81)	1.27 (1.65)
(7) EDDEP17	I felt sad	−0.54 (0.08)	1.10 (0.90)	1.16 (1.05)
(4) EDDEP07	I moved away from others (*)	−0.63 (0.08)	1.61 (4.84)	1.48 (2.86)
(28) EDANG29	I felt quick to anger	−0.66 (0.08)	1.34 (2.91)	1.42 (2.56)
(25) EDDEP12	I had mood swings	−1.14 (0.08)	1.30 (2.65)	1.43 (2.97)

Calibration: item calibration, i.e., item difficulty; SE: standard error; MnSq: Mean square; Zstd: Z-standardized. (*): misfitting items, i.e., items with infit MNSQ > 1.5 and/or outfit MNSQ > 2.0. Only item (4) EDDEP07 was misfitting.

## Data Availability

The data presented in this study are available on request from the corresponding author. The data are not publicly available due to restrictions, e.g., their containing information that could compromise the privacy of research participants.

## Supplementary Materials

The following supporting information can be downloaded at https://www.mdpi.com/article/10.3390/jcm14248774/s1. Supplementary Materials S1: Translation Process of PROMIS-Ca-D Depression; Supplementary Materials S2: Item Adjustments in the Item Bank Translation Process for PROMIS depression in cancer (PROMIS-Ca-D); Supplementary Materials S3: Methods of the Rasch analysis & Details on the results from the Rasch analysis.

## Abbreviations

The following abbreviations are used in this manuscript:

BMI	Body Mass Index
CTT	Classical Test Theory
DASS	Depression Anxiety and Stress Scale
DIF	Differential Item Functioning
FACIT	Functional Assessment of Chronic Illness Therapy
HADS	Hospital Anxiety and Depression Scale
IRT	Item Response Theory
MNSQ	Mean square
PCA	Principal Component Analysis
PCs	Principal components
PHQ-9	Patient Health Questionnaire—9 items
PROMIS	Patient-Reported Outcomes Measurement Information System
PROMIS-Ca-A	PROMIS Anxiety in Cancer questionnaire
PROMIS-Ca-D	PROMIS Depression in Cancer questionnaire
PROMs	Patient-reported outcome measures
RA	Rasch Analysis
RSM	Rating Scale Model
SD	Standard Deviation
SE	Standard Error
ZSTD	z-standardized statistics

## References

[1] Bray F., Laversanne M., Sung H., Ferlay J., Siegel R.L., Soerjomataram I., Jemal A. (2024). Global Cancer Statistics 2022: GLOBOCAN Estimates of Incidence and Mortality Worldwide for 36 Cancers in 185 Countries. CA A Cancer J. Clin..

[2] Nikbakhsh N., Moudi S., Abbasian S., Khafri S. (2014). Prevalence of Depression and Anxiety among Cancer Patients. Casp. J. Intern. Med..

[3] Smith H.R. (2015). Depression in Cancer Patients: Pathogenesis, Implications and Treatment (Review). Oncol. Lett..

[4] Tesio L., Scarano S., Hassan S., Kumbhare D., Caronni A. (2023). Why Questionnaire Scores Are Not Measures: A Question-Raising Article. Am. J. Phys. Med. Rehabil..

[5] Almigbal T.H., Almutairi K.M., Fu J.B., Vinluan J.M., Alhelih E., Alonazi W.B., Batais M.A., Alodhayani A.A., Mubaraki M.A. (2019). Assessment of Psychological Distress among Cancer Patients Undergoing Radiotherapy in Saudi Arabia. Psychol. Res. Behav. Manag..

[6] Clover K., Lambert S.D., Oldmeadow C., Britton B., King M.T., Mitchell A.J., Carter G. (2018). PROMIS Depression Measures Perform Similarly to Legacy Measures Relative to a Structured Diagnostic Interview for Depression in Cancer Patients. Qual. Life Res..

[7] Zigmond A.S., Snaith R.P. (1983). The Hospital Anxiety and Depression Scale. Acta Psychiatr. Scand..

[8] el-Rufaie O.E., Absood G. (1987). Validity Study of the Hospital Anxiety and Depression Scale among a Group of Saudi Patients. Br. J. Psychiatry.

[9] Lovibond P.F., Lovibond S.H. (1995). The Structure of Negative Emotional States: Comparison of the Depression Anxiety Stress Scales (DASS) with the Beck Depression and Anxiety Inventories. Behav. Res. Ther..

[10] Moussa M.T., Lovibond P., Laube R., Megahead H.A. (2017). Psychometric Properties of an Arabic Version of the Depression Anxiety Stress Scales (DASS). Res. Soc. Work. Pract..

[11] Kroenke K., Spitzer R.L., Williams J.B. (2001). The PHQ-9: Validity of a Brief Depression Severity Measure. J. Gen. Intern. Med..

[12] Spitzer R.L., Kroenke K., Williams J.B. (1999). Validation and Utility of a Self-Report Version of PRIME-MD: The PHQ Primary Care Study. Primary Care Evaluation of Mental Disorders. Patient Health Questionnaire. JAMA.

[13] Becker S., Al Zaid K., Al Faris E. (2002). Screening for Somatization and Depression in Saudi Arabia: A Validation Study of the PHQ in Primary Care. Int. J. Psychiatry Med..

[14] Al Harrasi S.M., Al-Awaisi H., Al Balushi M., Al Balushi L., Al Kharusi S. (2025). Fear of Cancer Recurrence and Its Association with Depressive Symptoms Among Adult Omani Female Breast Cancer Survivors. Int. J. Womens Health.

[15] Fekih-Romdhane F., Hakiri A., Fendri S., Balti M., Labbane R., Cheour M. (2021). Evaluation of Religious Coping in Tunisian Muslim Women with Newly Diagnosed Breast Cancer. J. Relig. Health.

[16] Cohen M., Gagin R., Cinamon T., Stein T., Moscovitz M., Kuten A. (2012). Translating “distress” and Screening for Emotional Distress in Multicultural Cancer Patients in Israel. Qual. Life Res..

[17] Ali A.M., Ahmed A., Sharaf A., Kawakami N., Abdeldayem S.M., Green J. (2017). The Arabic Version of The Depression Anxiety Stress Scale-21: Cumulative Scaling and Discriminant-Validation Testing. Asian J. Psychiatry.

[18] Cella D., Riley W., Stone A., Rothrock N., Reeve B., Yount S., Amtmann D., Bode R., Buysse D., Choi S. (2010). The Patient-Reported Outcomes Measurement Information System (PROMIS) Developed and Tested Its First Wave of Adult Self-Reported Health Outcome Item Banks: 2005–2008. J. Clin. Epidemiol..

[19] Cella D., Choi S., Garcia S., Cook K.F., Rosenbloom S., Lai J.-S., Tatum D.S., Gershon R. (2014). Setting Standards for Severity of Common Symptoms in Oncology Using the PROMIS Item Banks and Expert Judgment. Qual. Life Res..

[20] Rothmund M., Pilz M.J., Egeter N., Lidington E., Piccinin C., Arraras J.I., Grønvold M., Holzner B., van Leeuwen M., Petersen M.A. (2023). Patient-Reported Outcome Measures for Emotional Functioning in Cancer Patients: Content Comparison of the EORTC CAT Core, FACT-G, HADS, SF-36, PRO-CTCAE, and PROMIS Instruments. Psychooncology.

[21] Recklitis C.J., Blackmon J.E., Chang G. (2020). Screening Young Adult Cancer Survivors with the PROMIS Depression Short Form (PROMIS-D-SF): Comparison with a Structured Clinical Diagnostic Interview. Cancer.

[22] Lee M.K., Peipert J.D., Cella D., Yost K.J., Eton D.T., Novotny P.J., Sloan J.A., Dueck A.C. (2023). Identifying Meaningful Change on PROMIS Short Forms in Cancer Patients: A Comparison of Item Response Theory and Classic Test Theory Frameworks. Qual. Life Res..

[23] Tesio L., Caronni A., Kumbhare D., Scarano S. (2023). Interpreting Results from Rasch Analysis 1. The “Most Likely” Measures Coming from the Model. Disabil. Rehabil..

[24] Tesio L., Caronni A., Simone A., Kumbhare D., Scarano S. (2023). Interpreting Results from Rasch Analysis 2. Advanced Model Applications and the Data-Model Fit Assessment. Disabil. Rehabil..

[25] Aldhahi M.I., Bakhsh H.R., Bin Sheeha B.H., Alhasani R. (2024). Translation and Cross-Cultural Adaptation of an Arabic Version of PROMIS^®^ of Dyspnea Activity Motivation, Requirement Item Pool and Sleep-Related Impairments Item Bank. Health Qual. Life Outcomes.

[26] Bakhsh H.R., Aldhahi M.I., Aldajani N.S., Davalji Kanjiker T.S., Bin Sheeha B.H., Alhasani R. (2024). Arabic Translation and Rasch Validation of PROMIS Anxiety Short Form among General Population in Saudi Arabia. Behav. Sci..

[27] Bakhsh H.R., Aldajani N.S., Sheeha B.B., Aldhahi M.I., Alsomali A.A., Alhamrani G.K., Alamri R.Z., Alhasani R. (2023). Arabic Translation and Psychometric Validation of PROMIS General Life Satisfaction Short Form in the General Population. Healthcare.

[28] Garcia S.F., Cella D., Clauser S.B., Flynn K.E., Lad T., Lai J.-S., Reeve B.B., Smith A.W., Stone A.A., Weinfurt K. (2007). Standardizing Patient-Reported Outcomes Assessment in Cancer Clinical Trials: A Patient-Reported Outcomes Measurement Information System Initiative. J. Clin. Oncol..

[29] Teresi J.A., Ocepek-Welikson K., Kleinman M., Ramirez M., Kim G. (2016). Measurement Equivalence of the Patient Reported Outcomes Measurement Information System^®^ (PROMIS^®^) Anxiety Short Forms in Ethnically Diverse Groups. Psychol. Test. Assess. Model..

[30] Eremenco S.L., Cella D., Arnold B.J. (2005). A Comprehensive Method for the Translation and Cross-Cultural Validation of Health Status Questionnaires. Eval. Health Prof..

[31] Linacre J.M. (1994). Sample Size and Item Calibration Stability. Rasch Meas. Trans..

[32] Linacre J.M. (2023). Winsteps^®^ Rasch Measurement Computer Program User’s Guide.

[33] de Vet H.C.W., Terwee C.B., Mokkink L.B., Knol D.L. (2011). Validity. Measurement in Medicine: A Practical Guide.

[34] Borsboom D., Mellenbergh G.J., van Heerden J. (2004). The Concept of Validity. Psychol. Rev..

[35] Linacre J. (1999). Category Disordering (Disordered Categories) vs. Threshold Disordering (Disordered Thresholds). Rasch Meas. Trans..

[36] Linacre J. (2001). Category, Step and Threshold: Definitions & Disordering. Rasch Meas. Trans..

[37] Linacre J.M. (2010). Transitional Categories and Usefully Disordered Thresholds. Online Educ. Res. J..

[38] Linacre J.M. (2002). What Do Infit and Outfit, Mean-Square and Standardized Mean. Rasch Meas. Trans..

[39] Dimensionality: PCAR Contrasts & Variances: Winsteps Help. https://www.winsteps.com/winman/principalcomponents.htm.

[40] Linacre J.M. DIF—DPF—Bias—Interactions Concepts. https://www.winsteps.com/winman/difconcepts.htm.

[41] Tennant A., Pallant J. (2007). DIF Matters: A Practical Approach to Test If Differential Item Functioning Makes a Difference. Rasch Meas. Trans..

[42] Wright B.D., Stone M. (1999). Measurement Essentials.

[43] Caronni A., Picardi M., Scarano S., Rota V., Amadei M. (2025). Improving Single-Subject Change Assessment: Deriving the Minimal Detectable Change of Questionnaires’ Ordinal Scores from Rasch Analysis Measures. Disabil. Rehabil..

[44] Tennant A., Conaghan P.G. (2007). The Rasch Measurement Model in Rheumatology: What Is It and Why Use It? When Should It Be Applied, and What Should One Look for in a Rasch Paper?. Arthritis Care Res..

[45] Robitzsch A., Kiefer T., Wu M. (2025). TAM: Test Analysis Modules. https://cran.r-project.org/package=TAM.

[46] Vanhoutte E.K., Faber C.G., Van Nes S.I., Jacobs B.C., Van Doorn P.A., Van Koningsveld R., Cornblath D.R., Van Der Kooi A.J., Cats E.A., Van Den Berg L.H. (2012). Modifying the Medical Research Council Grading System through Rasch Analyses. Brain.

[47] Tennant A., Küçükdeveci A.A. (2023). Application of the Rasch Measurement Model in Rehabilitation Research and Practice: Early Developments, Current Practice, and Future Challenges. Front. Rehabil. Sci..

[48] Asamoah N.A.B., Turner R.C., Lo W.-J., Crawford B.L., McClelland S., Jozkowski K.N. (2025). Evaluating Item Response Format and Content Using Partial Credit Trees in Scale Development. J. Surv. Stat. Methodol..

[49] Adams R.J., Wu M.L., Wilson M. (2012). The Rasch Rating Model and the Disordered Threshold Controversy. Educ. Psychol. Meas..

[50] Andrich D. (2013). An Expanded Derivation of the Threshold Structure of the Polytomous Rasch Model That Dispels Any “Threshold Disorder Controversy”. Educ. Psychol. Meas..

[51] Mallinson T., Kozlowski A.J., Johnston M.V., Weaver J., Terhorst L., Grampurohit N., Juengst S., Ehrlich-Jones L., Heinemann A.W., Melvin J. (2022). Rasch Reporting Guideline for Rehabilitation Research (RULER): The RULER Statement. Arch. Phys. Med. Rehabil..

[52] Van de Winckel A., Kozlowski A.J., Johnston M.V., Weaver J., Grampurohit N., Terhorst L., Juengst S., Ehrlich-Jones L., Heinemann A.W., Melvin J. (2022). Reporting Guideline for RULER: Rasch Reporting Guideline for Rehabilitation Research: Explanation and Elaboration. Arch. Phys. Med. Rehabil..

[53] HealthMeasures (2023). Anxiety Measure Differences: A Brief Guide to Differences Between the PROMIS^®^ Anxiety Instruments.

[54] HealthMeasures (2023). Depression Measure Differences: A Brief Guide to Differences Between the PROMIS^®^ Depression Instruments.

[55] Caronni A., Scarano S. (2024). Generalisability of the Barthel Index and the Functional Independence Measure: Robustness of Disability Measures to Differential Item Functioning. Disabil. Rehabil..

[56] de Castro N.F.C., de Melo Costa Pinto R., da Silva Mendonça T.M., da Silva C.H.M. (2020). Psychometric Validation of PROMIS^®^ Anxiety and Depression Item Banks for the Brazilian Population. Qual. Life Res..

[57] Fisher W.P. (2010). IRT and Confusion about Rasch Measurement. Rasch Meas. Trans..

[58] Negrini S., Zaina F., Buyukaslan A., Fortin C., Karavidas N., Kotwicki T., Korbel K., Parent E., Sanchez-Raya J., Shearer K. (2023). Cross-Cultural Validation of the Italian Spine Youth Quality of Life Questionnaire: The ISYQOL International. Eur. J. Phys. Rehabil. Med..

