# Supplementary material for: Validity of the Arabic Version of the PROMIS Anxiety and PROMIS Depression in Cancer Questionnaires: Measuring Depression and Anxiety in Oncologic Patients in Saudi Arabia—A Rasch Analysis Study
Source: J Clin Med. 2025 Dec 11;14(24):8774. doi: 10.3390/jcm14248774 (PMC12734167; doi:10.3390/jcm14248774)
Supplement: Supplementary file 1 [file jcm-14-08774-s001.zip › promisAnxDep - supplMat1 - 2025 11 26.pdf]

SUPPLEMENTARY MATERIALS 1

Construct validity of the Arabic Version of the PROMIS Anxiety and PROMIS Depression in Cancer questionnaires: measuring Depression and Anxiety in oncologic patients in Saudi Arabia – a Rasch analysis study.

Hadeel R, Bakhsh[1]*, Bodor H. Bin Sheeha[1], Luigi Tesio[2], Anna Simone[2], Stefano Scarano[2], Monira I. Aldhahi[1], Nouf Alowain[1], Ghada A. bin Dayel, Rehab Alhasani[1], Antonio Caronni[2,3]

[1] Department of Rehabilitation Sciences, College of Health and Rehabilitation Sciences, Princess Nourah bint Abdulrahman University, Riyadh, Saudi Arabia.

[2] Department of Neurorehabilitation Sciences, IRCCS Istituto Auxologico Italiano, Milano, Italy

[3] Department of Biomedical Sciences for Health, University of Milan, Italy

*** Corresponding Author**

Hadeel R. Bakhsh Hrbakhsh@pnu.edu.sa

Translation Process of PROMIS-Ca-D Depression

The translation was conducted according to the **Functional Assessment of Chronic Illness Therapy (**FACIT) translation protocol by a team of linguists, translators, proofreaders, and authors (BBS, HRB, MIA, and RA) **[40-42].** Translation into Arabic involved the following steps :

1. Two independent professional translators, who were native Arabic speakers, conducted forward translations from English into Arabic.
2. A third translator, native Arabic speaker, checked the translated versions and created a provisional Arabic version.
3. Next, a native English speaker (i.e. a fourth translator) back translated the Arabic version into English.
4. The translation project manager compared the original English and back-translated versions to identify possible differences.
5. In the last stage, in case of critical issues highlighted by the translation project manager for any item, three bilingual translation specialists selected the best translation option and offered a few alternatives.
6. The FACIT team, in collaboration with the PROMIS Statistical Centre, made the final assessment and performed consistency checks, again between the questionnaire back translated from Arabic to English and the source English version.

Cognitive interviews and pilot testing were carried out by the authors (BHB, HRB, MIA, and RA), who were proficient in these assignments in line with our previous studies [43-45].

FACIT Translation Process flowchart

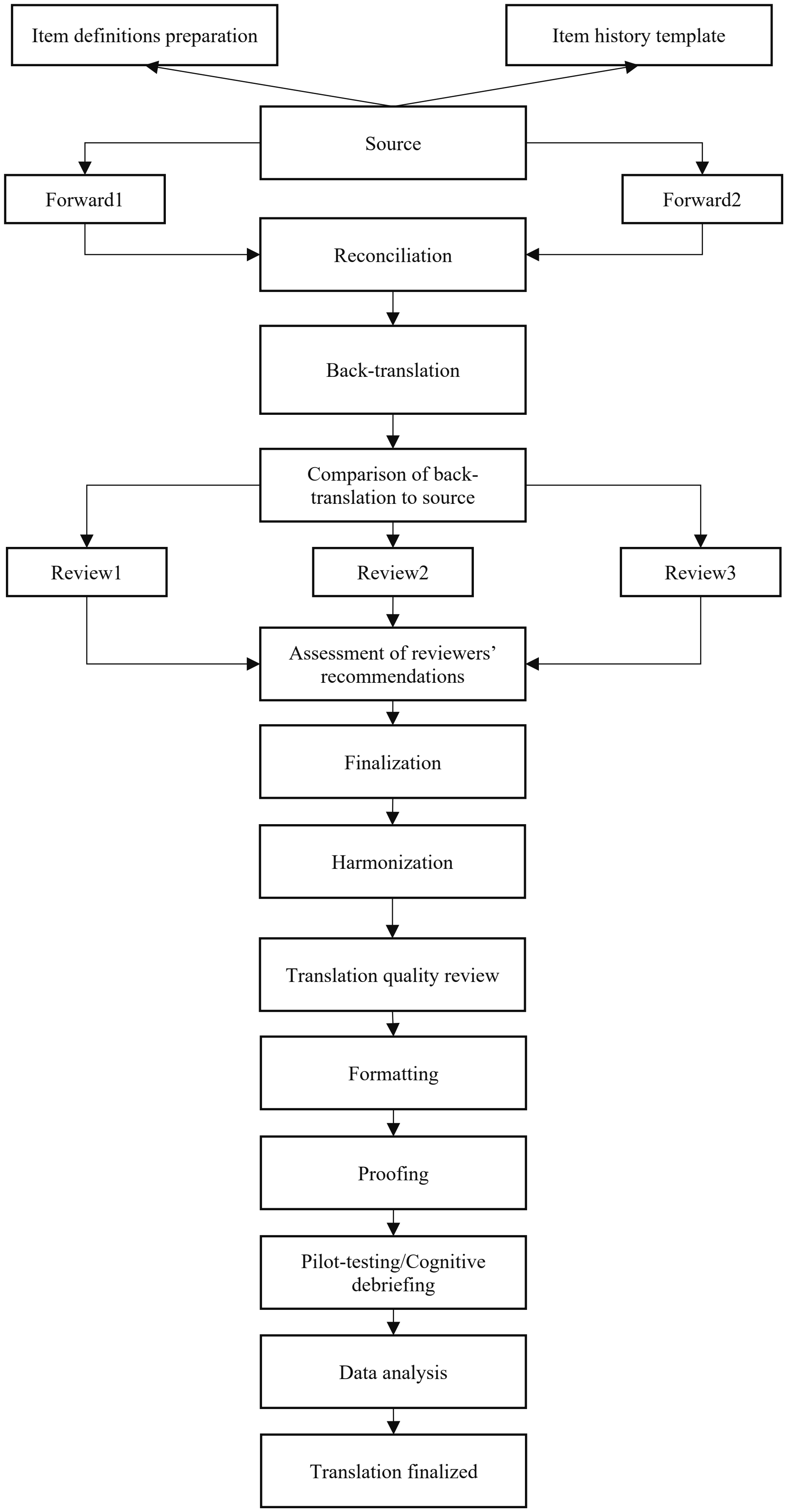

Table 1 The FACITtrans Arabic translation team

Role*	Qualification	Title & Profession
Translation Account Manager FACITtrans	MBA	Director, PROMIS Lead
Translation Project Coordinator FACITtrans	BA	Senior COA Translations Manager - Life Sciences, PROMIS Specialist
Translation Project Manager FACITtrans	BA, Spanish Linguistics	Senior COA Translation Project Manager – Life Sciences
Forward 1	BA, Languages and Translation, Simultaneous Interpretation (English < >Arabic)	Senior Translator, Copywriter & Proofreader Professional and Translator Interpreter
Forward 2	MA, Linguistics	Professional Translator and Interpreter
Reconciler/Proofreader	Ph.D., Linguistics	Professional Linguist and Translator
Back Translator	MA, Diplomacy<br>BA, Medical Technology	Professional Translator<br>16 years full immersion in Arabic public school system and 3.5 years of undergraduate education (nursing) at King Abdul Aziz University, Jeddah Saudi Arabia
Reviewer 1	Ph.D., Linguistics	Linguist and researcher<br>Pragmatics, sociolinguistics, discourse analysis, ideology, identity, and translation studies
Reviewer 2	Ph.D., Linguistics	Professional Linguist and Translator
Reviewer 3, Language Coordinator, Proofreader 1	DDS<br>MA, Biblical Studies	Professional Translator and Interpreter<br>Close to 30 years' experience specializing in medical, legal and religious translation

Reference:

[1] Cleanthous S, Barbic SP, Smith S, Regnault A. Psychometric performance of the PROMIS® depression item bank: a comparison of the 28-and 51-item versions using Rasch measurement theory. Journal of Patient-Reported Outcomes 2019;3:1-12.

[2] Bonomi AE, Cella DF, Hahn EA, Bjordal K, Sperner-Unterweger B, Gangeri L, Bergman B, Willems-Groot J, Hanquet P, Zittoun R. Multilingual translation of the Functional Assessment of Cancer Therapy (FACT) quality of life measurement system. Quality of Life Research 1996;5:309-20.

[3] Eremenco SL, Cella D, Arnold BJ. A Comprehensive Method for the Translation and Cross-Cultural Validation of Health Status Questionnaires. Evaluation & the Health Professions 2005;28:212-32.

[4] Bakhsh HR, Aldajani NS, Sheeha BB, Aldhahi MI, Alsomali AA, Alhamrani GK, Alamri RZ, Alhasani R, editor^editors. Arabic Translation and Psychometric Validation of PROMIS General Life Satisfaction Short Form in the General Population. Healthcare; 2023: MDPI. p 3034.
