# Supplementary material for: Validity of the Arabic Version of the PROMIS Anxiety and PROMIS Depression in Cancer Questionnaires: Measuring Depression and Anxiety in Oncologic Patients in Saudi Arabia—A Rasch Analysis Study
Source: J Clin Med. 2025 Dec 11;14(24):8774. doi: 10.3390/jcm14248774 (PMC12734167; doi:10.3390/jcm14248774)
Supplement: Supplementary file 1 [file jcm-14-08774-s001.zip › promisAnxDep - supplMat2 - 2025 11 26.pdf]

SUPPLEMENTARY MATERIALS 2

Construct validity of the Arabic Version of the PROMIS Anxiety and PROMIS Depression in Cancer questionnaires: measuring Depression and Anxiety in oncologic patients in Saudi Arabia – a Rasch analysis study

Hadeel R, Bakhsh[1]*, Bodor H. Bin Sheeha[1], Luigi Tesio[2], Anna Simone[2], Stefano Scarano[2], Monira I. Aldhahi[1], Nouf Alowain[1], Ghada A. bin Dayel[1], Rehab Alhasani[1], Antonio Caronni[2,3]

[1] Department of Rehabilitation Sciences, College of Health and Rehabilitation Sciences, Princess Nourah bint Abdulrahman University, Riyadh, Saudi Arabia.

[2] Department of Neurorehabilitation Sciences, IRCCS Istituto Auxologico Italiano, Milano, Italy

[3] Department of Biomedical Sciences for Health, University of Milan, Italy

*** Corresponding Author**

Hadeel R. Bakhsh Hrbakhsh@pnu.edu.sa

Item Adjustments in the Item Bank Translation Process for PROMIS depression in cancer (PROMIS-Ca-D)

Source item	English equivalent	Translation issues/ Cognitive debriefing feedback	Reasons for adaptation
PROMIS Item Bank Depression	**EDANG09- I felt angry**<br><br>**EDANG29- I felt irritable**	EDANG09 I felt angry شعرت بالغضب<br><br>EDANG29 I felt irritable شعرت باني سريع الغضب<br><br>these two items were translated to the same meaning, "I felt angry: translated right; I felt irritable: it supposed to be translated to<br>شعرت باني سريع الانزعاج	Participants found item EDANG09 similar to EDANG29, to make it more clear<br><br>Item kept as it is<br>EDANG09,<br>I felt angry شعرت بالغضب<br><br>And the item EDANG29 modified to 'I felt irritable'<br>شعرت باني سريع الانزعاج (سريع الغضب)
