# Supplementary material for: Validity of the Arabic Version of the PROMIS Anxiety and PROMIS Depression in Cancer Questionnaires: Measuring Depression and Anxiety in Oncologic Patients in Saudi Arabia—A Rasch Analysis Study
Source: J Clin Med. 2025 Dec 11;14(24):8774. doi: 10.3390/jcm14248774 (PMC12734167; doi:10.3390/jcm14248774)
Supplement: Supplementary file 1 [file jcm-14-08774-s001.zip › promisAnxDep - supplMat3 - 2025 11 26.pdf]

SUPPLEMENTARY MATERIALS 3

Validity of the Arabic Version of the PROMIS Anxiety and PROMIS Depression in Cancer questionnaires: measuring Depression and Anxiety in oncologic patients in Saudi Arabia – a Rasch analysis study.

Hadeel R, Bakhsh[1]*, Bodor H. Bin Sheeha[1], Luigi Tesio[2], Anna Simone[2], Stefano Scarano[2], Monira I. Aldhahi[1], Nouf Alowain[1], Ghada A. bin Dayel[1], Rehab Alhasani[1], Antonio Caronni[2,3]

[1] Department of Rehabilitation Sciences, College of Health and Rehabilitation Sciences, Princess Nourah bint Abdulrahman University, Riyadh, Saudi Arabia.

[2] Department of Neurorehabilitation Sciences, IRCCS Istituto Auxologico Italiano, Milano, Italy

[3] Department of Biomedical Sciences for Health, University of Milan, Italy

*** Corresponding Author**

Hadeel R. Bakhsh Hrbakhsh@pnu.edu.sa

Abbreviations

DIF = Differential Item Functioning

MNSQ = mean square

PCA = Principal Component Analysis

PROMIS-Ca-D = PROMIS depression in cancer

PROMIS-Ca-A = PROMIS anxiety in cancer

RA = Rasch Analysis

ZSTD = standardised statistics

Appendix 1 – Methods: the Rasch analysis

The Rasch analysis (RA; rating scale model) was used to assess the construct validity and the reliability of the Arabic versions of the PROMIS depression in cancer (PROMIS-Ca-D) and PROMIS anxiety in cancer (PROMIS-Ca-A) questionnaires.

The following questionnaire's psychometric features were assessed: 1) categories functioning, 2) data-model fit, 3) dimensionality, 4) questionnaire maps, 5) Differential Item Functioning and 6) persons' measure reliability.

1. Categories functioning.

The Rasch analysis assumes that items have ordered categories. While there is full agreement in the Rasch analysis community about this prerequisite to the analysis, no consensus appears to have been reached about the method to empirically assess if data complies with the ordered categories' requirement [1,2].

Technically speaking, verifying that categories are ordered is verifying that a monotonic relationship exists between the quantity measured with the questionnaire and the categories' numerals. There are two ways (which can lead to contrasting results) to assess category order in the RA, and both are applied in this work.

According to Linacre [3], when applied to the PROMIS-Ca-D and PROMIS-Ca-A questionnaires, "ordered categories" mean that those respondents choosing higher categories numerals suffer, *on average,* higher anxiety and depression levels. According to Andrich [2], the categories' functioning is assessed by evaluating the order of *modal* (i.e. Andrich) thresholds. A modal threshold is the point along the construct line (here, depression and anxiety) where the chance of being scored in one of two adjacent categories is equal. For the current study, modal thresholds are disordered if, for example, the threshold between categories 2 and 3 is on the left of that between categories 1 and 2 along the anxiety or depression continuum. Such a finding would indicate that the anxiety (or depression) level for which categories 1 and 2 are equally probable is higher than that for which categories 2 and 3 are equally probable.

A true controversy about how the category's order should be assessed empirically has been pointed out [1,2,4] with eminent scholars on both sides of the argument.

Regarding this issue, it is just mentioned here that the relationship between modal thresholds and categories functioning is more intricate than it might seem at first glance. Simulations highlight that ordered categories can show disordered modal thresholds when a category is rarely chosen. To complement this, categories that have been artificially disordered can have ordered modal thresholds. So, while categories malfunctioning is a cause of disordered thresholds, this does not seem to be the only one [5].

Given the above, for the current study, questionnaires are considered robust to some modal threshold disordering. Therefore, we continue the analysis in the main text provided that the categories' average measures are ordered. However, for completeness, in Appendix 3 in these Supplementary Materials, an additional analysis is also reported in which the categories are collapsed if disordered modal thresholds are found. Category collapsing, i.e. relabelling two (or more) consecutive categories in a single new category, is a customary procedure to solve thresholds' disordering.

2. *Data-model fit*

Infit and outfit mean square (MNSQ) and z-standardised (ZSTD) statistics were calculated to investigate item fit to the Rasch (rating-scale) model.

The size of the data departure from the model's expectation is quantified with the MNSQ, a statistic calculated from the squared standardised residuals. The ZSTD statistic can be considered a significance test of the null hypothesis of no difference between data and model. In plain words, MNSQ works as an effect size of the distance between data and the model. If this departure is statistically significant, it is tested with the ZSTD.

Considering here only the simpler case of the outfit MNSQ and using a simplified notation, the squared residual y^2 is given by the difference between the observed score X and the expected score E from the model [6]:

$$y^2 = (X - E)^2.$$

This squared residual is then standardised to the variance V of the expected value E to obtain the squared standardised residual z^2:

$$z^2 = y^2 / V$$

The outfit MNSQ corresponds to the mean of these squared standardised residuals:

$$\text{Outfit MNSQ} = (\sum z^2) / N.$$

Now, let's take the square root of the squared standardised residual z^2:

$$z = y / V^{0.5}$$

The difference y is expressed as a certain number of standard deviations, similar to what happens with effect size indices (e.g. Cohen's d [7]).

Being built on squared residuals, it is not surprising that, given an MNSQ and its degrees of freedom (df), the MNSQ can be easily turned into a chi-squared statistic:

$$\text{Chi-squared} = x^2 = \text{MNSQ} \times \text{df}.$$

From the chi-squared statistics x^2 and the df, it is possible to run the chi-squared (χ^2) test to test the null hypothesis: *"There is no difference between the data and model's expectation"* (i.e. the distance between data and model is nil) [8]. While a table or software is enough to derive the probability that the null hypothesis is true, given the observed chi-square and degrees of freedom, it is possible to obtain this probability even more straightforwardly.

The cube root of a chi-square statistic x^2 divided by its df is approximately normally distributed, a transformation of x^2 named after Wilson and Hilferty [9]. Since the mean and the standard deviation of the distribution of this transformed x^2 can be calculated from the x^2 df, it is possible to transform further x^2 into a normal distribution with a mean of 0 and standard deviation equal to 1 [9]. Therefore, the Wilson and Hilferty transformation eventually turns x^2 into a standard score so that p-values can be derived seamlessly without referring to df values and, hence, to tables and software.

The ZSTD is obtained by applying the Wilson and Hilferty transformation to the MNSQ statistics. Being the standard score of a normal distribution, if ZSTD > 1.96, the residuals significantly differ from zero, and data significantly depart from the model's expectation at $p < 0.05$. In this sense, ZSTD can be said to be the significance test of MNSQ: the MNSQ gives the size of the departure, and the ZSTD the significance of this departure.

The main difference between infit and outfit statistics is that the latter (with “outfit” for “outlier sensitive”) is more sensitive to high-ability respondents unexpectedly failing easy items (and low-ability respondents passing difficult items). Infit statistics (with “infit” for “inlier sensitive”) are more sensitive to unexpected responses from respondents whose ability level matches item difficulty.

As per cutoff values, in this study, infit MNSQ indicated poor data fit if > 1.5. An item was considered misfitting if its outfit MNSQ was > 2.0. These cutoff values, on the liberal side, consider that items with MNSQ > 2.0 degrade questionnaire measures, while items with MNSQ between 1.5 and 2.0 are unproductive but do not distort measurements [3,10].

Different thresholds were used for infit and outfit MNSQ because of the outfit MNSQ sensitivity to outliers. Regarding this, it is a common experience at the analysis stage that large outfit MNSQ values can be found in the eventuality of a few unexpected responses, which can happen for inattention, fatigue or guessing and not necessarily flagging a substantial ambiguity of the item [11].

To get an idea of the functioning of an item with a large infit MNSQ, think of an item of medium difficulty correctly passed by high-ability respondents and failed by low ability levels. However, respondents measuring slightly above the item’s calibration fail the item more often than expected, and those measuring slightly below the item’s calibration pass it more often than predicted by the model. In this example, the total residuals, i.e. the MNSQ, increase if enough participants whose measure is not very distant from the item’s calibration give unexpected responses to the item. On the contrary, about outfit MNSQ, a high-ability respondent failing a straightforward item produces a large residual, rapidly inflating the MNSQ.

3. *Dimensionality assessment*

The RA assumes that questionnaires’ total scores are unidimensional, i.e., they reflect the quantity of a single variable.

The Principal Component Analysis (PCA) was performed on the model’s standardised residuals to ascertain whether the Rasch model’s assumption of scale unidimensionality is satisfied. As customary [3], hidden variables potentially affecting item scores in addition to the variable modelled by the Rasch model are pointed out by one or more principal components (PCs) with eigenvalue > 2.0.

Regarding this eigenvalue threshold, it should be remembered that the PCA examines model residuals to discover item correlations, and the smallest set of items that can correlate consists precisely of two items.

In the framework of the PCA of the Rasch model’s residuals, the eigenvalues quantify the strength of the additional variable in terms of the number of items whose scores are affected by this variable [3]. For example, a principal component with an eigenvalue of 3.82 affects the score of up to four items. Therefore, the larger the eigenvalue, the more numerous the items whose scores correlate because of an additional variable, constituting a true sub-questionnaire within the primary questionnaire. Hence, the larger the eigenvalue, the more dangerous the hidden variable will eventually become.

In the current work, a two-step approach was adopted to assess multidimensionality.

First, the eigenvalues are calculated and interpreted as described above. Next, in the case PCs with eigenvalue > 2.0 are found, the second step assesses whether this multidimensionality causes a substantial malfunctioning of the interval (logit) measures extracted from the questionnaires’ total scores.

Whether multidimensionality harms measurements is tested as done elsewhere [3,10]. Items are grouped into clusters according to their loading on the PC: cluster 1 and 3 items have the largest positive and negative loadings, respectively, while cluster 2 items load low on the component. So, the score to cluster 2 items can be assumed only to reflect the Rasch dimension. In contrast, clusters 1 and 3 items are affected by the Rasch dimension and, oppositely, by the additional variable indicated by the principal component. Participants are measured with the three clusters, and agreement between the three sets of measures is tested using ANOVA and Pearson's correlation. Satisfactory agreement between clusters 1 and 2 and clusters 3 and 2 is evidence that the Rasch dimension, rather than the additional one, is the most decisive in determining the scores of clusters 1 and 3 items.

4. *Items, thresholds and persons maps*

Items, thresholds and person maps report the items' and thresholds' calibrations and persons' measures (in logits) graphically along the line representing the construct (i.e. here, depression and anxiety).

Items and threshold maps allow for an adequate assessment of how questionnaire items assess the construct of interest. Large gaps between items or thresholds indicate an unexplored range of the variable, flagging a problem with construct validity [12].

Comparing the items and persons maps is an easy way to assess how much items' calibrations match participants' ability, i.e., item-person targeting. Items are "on target" with the sample when the person's mean ability is within 0.5 logits of the item mean, zero logits by convention [3].

5. *Differential Item Functioning*

Differential Item Functioning (DIF) corrupts an item if the item does not work the same psychometrically in respondents from different groups (e.g., young and old persons, persons with different diagnoses). More precisely, an item has DIF if two respondents with the same level of the variable of interest, i.e., the same total score (e.g. with the same depression or anxiety level) but belonging to different groups score differently on this item. In this scenario, the item is harder to pass (or endorse) in one group than another and is unfair to respondents in this group.

Assessing DIF is another method for detecting multidimensionality. The score of an item with DIF depends on the quantity of the "Rasch" variable and the variable splitting the respondents into groups (e.g. age and cancer diagnosis).

Uniform DIF was tested for the following groups:

1. sex (male vs female),
2. age (< 40 years, ≥ 40 & < 60 years, class 2: ≥ 60 years),
3. education level (uneducated, primary or secondary school, high school, university),
4. pre-existing mental disorder (yes vs no),
5. social status (married vs alone),
6. cancer localisation (single vs. multiple), physically active (yes vs no),
7. time from cancer diagnosis (≤ 1 year, > 1 & ≤ 5 years, > 5 years) and
8. employment (unemployed vs student or worker vs retired).

Similarly to the dimensionality assessment, a two-step procedure was applied to the DIF assessment. First, it was evaluated if DIF was present in mathematical terms. Second, it was assessed if DIF was large enough to cause severe

malfunctioning of the measures obtained from the questionnaire's total score. Similar to what was done in the dimensionality evaluation, it was verified whether DIF was large enough to matter practically.

DIF was present for an item if the item calibrations were significantly different ($p < 0.05$, two-sided t-test) in the two groups of respondents, and this difference was > 0.5 logits [13]. If DIF corrupted more items for the same variable, the impact of DIF on the questionnaire measures was tested as done elsewhere.

Say DIF is found for pre-existing mental disorders with a bunch of items with a lower calibration in respondents with a history of previous mental distress compared to those without. A set of "pure" items is isolated by removing all the items with DIF for pre-existing mental disorders [14,15].

Participants are measured with the set of pure items and the entire questionnaire, i.e. including the pure items and the items corrupted by DIF. Since each person's measure comes with a standard error, in the RA measurement framework, it is possible to run t-tests to assess if two measures of a single person are statistically different at $p < 0.05$.

In line with the procedure by Smith [15], the number of persons measuring differently with the pure items and the total questionnaire is counted and expressed in percentage of the participants' sample. If this percentage is not significantly larger than the customary 5% threshold (exact binomial test, 95% confidence interval), the two questionnaires, the full one and that consisting of DIF-free, pure items only, measure single persons substantially the same. If this is the case, even if DIF is mathematically present, it is of no harm for measures from a practical point of view.

Regarding the impact of DIF, it is also worth mentioning that measures from questionnaire total scores show remarkable robustness even to a large DIF of a few items [16]. For example, in several instances, DIF between 0.5 and 1 logit is not strong enough to cause the malfunctioning of the measures from the questionnaire total scores.

6. *Person measure reliability*

Rasch's person separation reliability is given by one minus the ratio of the root mean square of person measures error to the sample person measure variance [17–19]. It is, therefore, a ratio of true variance to observed variance, aligning with the classical definition of reliability in psychometrics.

From this reliability index, the number of strata is obtained, representing the levels of depression or anxiety a questionnaire can discern at the single-subject level. As with any reliability index, Rasch's reliability can be interpreted as: > 0.90, appropriate for individual decisions; > 0.70, appropriate for group decisions [20].

7. *Score-to-measure conversion*

When a questionnaire passes the Rasch analysis, interval measures can be extracted from the questionnaire's total ordinal score. The score-to-measure conversion is commonly provided in a tabular form where each table row reports a questionnaire's total score value, the corresponding interval measure and its standard error of the measure.

Appendix 1.1 – Assessment of Sample Size Adequacy for Stable Item Calibration Estimates

The results presented here are based on a sample of 213 participants (from 250 individuals originally invited). In the tradition of Rasch analysis, this sample size is considered sufficient for reliable item calibrations, even in high-stakes contexts [21].

However, following the discussion with the reviewers of the first manuscript version, we decided to conduct a series of statistical simulations to assess the actual stability of the item calibrations for the questionnaires tested here, given the sample size used. In this context, assessing stability means evaluating the margin within which these calibrations might plausibly vary if the analysis were replicated in a larger sample of participants. The underlying rationale was as follows.

Random samples ranging from 25 to 200 participants, in 25-participant increments (i.e., 25, 50, 75...), were drawn without replacement from the full sample of 213 participants. For each of these samples, the Rasch analysis (Rating Scale Model) was performed, and the item calibrations were extracted. For each sample size (25, 50, ..., 200), the extraction process was iterated 250 times. Therefore, for each sample size, 250 different calibration estimates were available for each item in the questionnaire.

For each item, the 2.5th and 97.5th percentiles of its calibration were calculated (given the series of 250 estimates obtained from the simulation iterations). The width of the interval between the 2.5th and 97.5th percentiles reflects the stability (or precision) of the item calibration, given a specific sample size.

The graph in Figure S1A shows the results of this simulation for the PROMIS-Ca-A questionnaire. The horizontal axis reports the sample size of the different samples drawn from the full sample. The vertical axis reports the width of the 97.5th – 2.5th percentile range for each of the 22 items that constitute the PROMIS-Ca-A (one dot per item). It is immediately evident that as the sample size increases, this calibration range decreases. This reduction is clearly non-linear. This non-linearity indicates that, as expected, as the sample size increases, the item calibrations will be more and more stable, meaning that for a given sample size, the calibration of an item will change less and less from sample to sample.

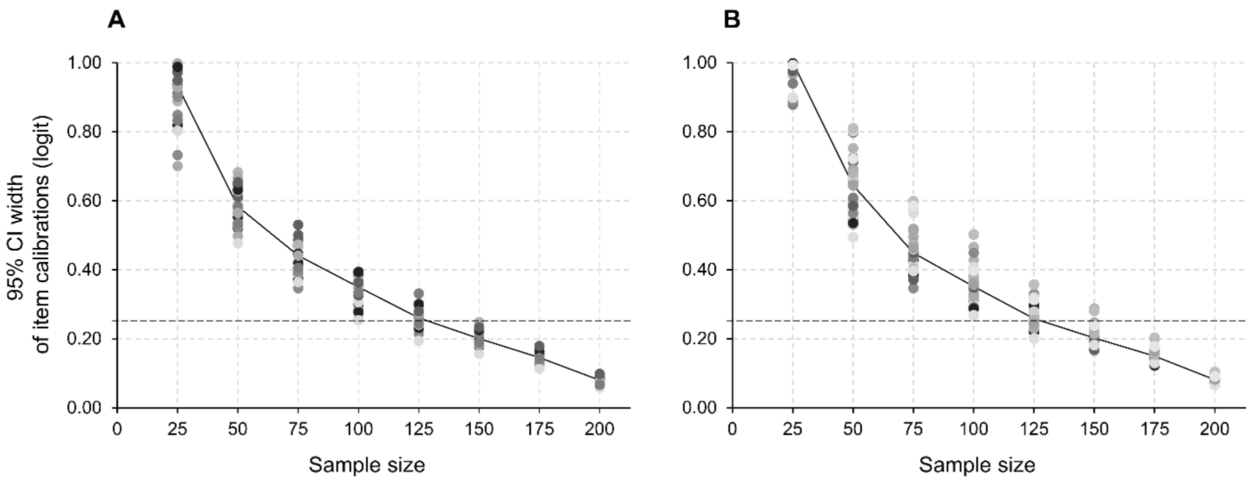

Figure S1. Stability of PROMIS-Ca-A and PROMIS-Ca-D item calibrations across varying sample sizes. (A) The graph reports the width of the 95% confidence interval (97.5th – 2.5th percentile) for the item calibration estimates across the 250 resampling iterations, for each of the 22 items that constitute the PROMIS-Ca-A (one dot per item). Samples ranging from 25 to 200 participants were considered, in increments of 25. The solid line connects the median values of the 22 items. The dashed horizontal line represents 0.25 logits. (B) Shows the same analysis for the PROMIS-Ca-D questionnaire, which consists of 30 items.

For a sample size of N=150, the item calibrations of the PROMIS-Ca-A questionnaire show a [97.5th – 2.5th percentile] range width between a minimum of 0.16 logits (for the most stable item) and a maximum of 0.25 logits (for the least stable item). This means that, in the worst-case scenario (i.e., for the least stable item), we can be 95% confident that the population calibration of an item lies within the interval comprising the observed calibration ± 0.125 logits.

As the sample size increases, the precision of the item calibration estimates further increases (Figure S1A), but the fact remains that even at N=150, this estimate is sufficiently precise. We consider this precision to be adequate, relying on the well-established convention in Rasch analysis that a difference between two measures or calibrations must be at least 0.50 logits to be considered substantially relevant [16]. With a calibration uncertainty of ± 0.125 logits, this sample size allows for the reliable distinction of two item calibrations as significantly different if they are separated by at least 0.25 logits. This implies that the resolution of the item map is double the conventional threshold of 0.5 logits. Moreover, given that our actual sample size (N=213) exceeds this threshold, we can expect even greater precision in our item calibration estimates.

Based on this simulation study, we propose that the sample recruited here (N=213) is sufficiently large that it would be surprising (or highly unlikely) if—as is also indicated by previous studies [21]—adding more participants were to result in dramatically different item calibrations (and thus, ultimately, person measures).

Similar results were found for PROMIS-Ca-D (Figure S1B). Compared to PROMIS-Ca-A, the stability appears slightly lower, presumably because PROMIS-Ca-D has a higher number of items (30 vs 22) and thus a greater number of parameters to estimate. Consequently, the ratio between the number of observations and the number of estimated parameters (item and threshold) is less favorable, which translates into lower stability of the estimates.

Appendix 2 – Details on the results from the Rasch analysis

Appendix 2.1 – Dimensionality of the PROMIS Anxiety and PROMIS Depression in Cancer Questionnaires

Regarding the PROMIS Anxiety in Cancer (PROMIS-Ca-A) questionnaire, the Principal Component Analysis (PCA) showed two principal components (PCs) with an eigenvalue > 2.0. Notably, the eigenvalues were just above this threshold (2.46 and 2.24 for PC1 and PC2, respectively), and, in terms of item loading, only one item per PC had a loading ≥ 0.6 (Table S1).

This last finding indicates that the hidden variables highlighted by the two PCs affected single items rather than several items simultaneously, as would be the case with typical multidimensional questionnaires.

Table S1. Principal Component Analysis of the PROMIS Anxiety in Cancer and PROMIS Depression in Cancer questionnaires.

		Positive loading			Negative loading		
	**PC**	**Item**	**Cluster**	**Loading**	**Item**	**Cluster**	**Loading**
**PROMIS-Ca-A**	1	EDANX02	1	0.65	-	-	-
	2	EDANX09	1	0.60	-	-	-
**PROMIS-Ca-D**	1	EDDEP41	1	0.63	-	-	-
	2	-	-	-	-	-	-
	3	-	-	-	-	-	-

PROMIS-Ca-A: PROMIS Anxiety in Cancer questionnaire; PROMIS-Ca-D: PROMIS Depression in Cancer questionnaire; PC: principal component; only the items with a loading ≥ 0.6 are reported.

Regarding the PROMIS Depression in Cancer (PROMIS-Ca-D) questionnaire, three PCs had eigenvalue > 2 (2.67, 2.13, and 2.05), but there was a PC only (PC1) with an item only with loading > 0.6 (Table S1).

Even if, based on the above, the impact of multidimensionality seems minor, its effects on the PROMIS-Ca-A and PROMIS-Ca-D measures were assessed further.

Figure S2 shows the sample mean measures returned by the PROMIS-Ca-A items, grouped into three clusters per their loading on the PC1 and PC2, i.e. the two PCs with eigenvalues > 2.0.

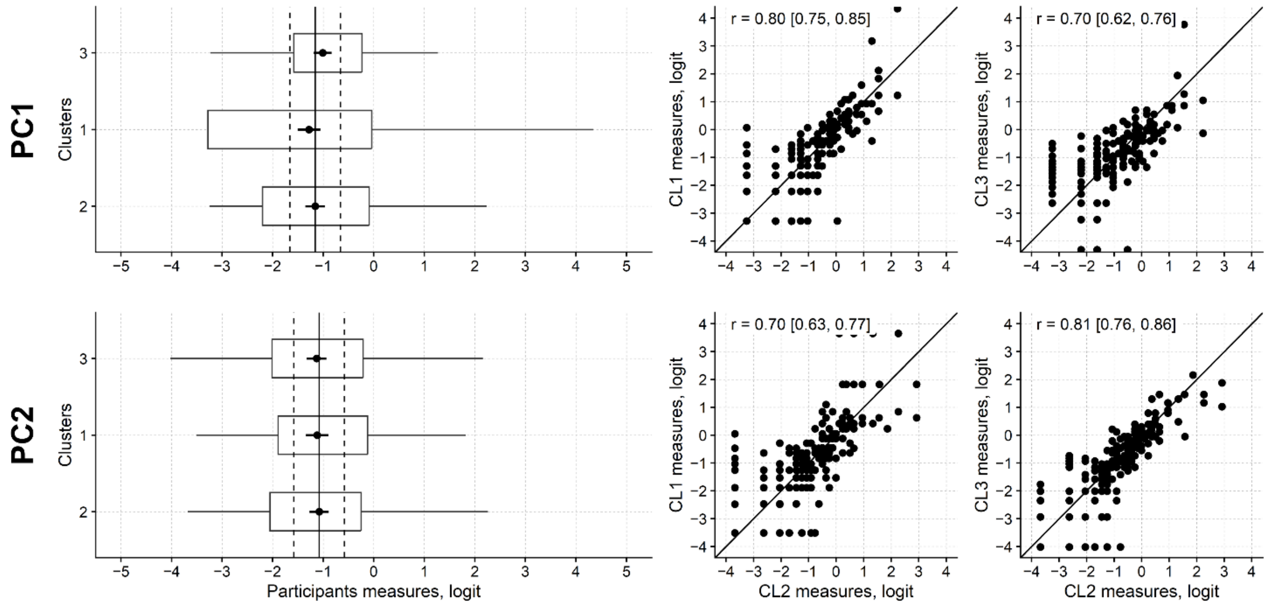

Figure S2: Dimensionality analysis of the PROMIS Anxiety in Cancer questionnaire. The boxplots show the distribution of the participants measures from clusters 1, 2 and 3 items. Cluster 1 items had a large and positive loading on the PC, cluster 3 had a large and negative loading and cluster 2 had a low loading. Hence, cluster 2 items can be considered unaffected by the additional variable highlighted by the PC. Central dot in the box of the boxplot: participants' mean measure and its 95% confidence interval (horizontal bars). Vertical continuous line: cluster 2 mean measure; vertical dashed lines: cluster 2 mean measure plus or minus 0.5 logits. Only the PC with an eigenvalue > 2.0 are considered. PC: principal component; CL: cluster; r: coefficient [and 95% confidence interval] from Pearson's correlation.

Table S2 reports the ANOVA comparing the participants' measures from the cluster of items.

Table S2. ANOVA comparing the respondents' measures from items cluster 1, 2 and 3 of the PROMIS Anxiety in Cancer and PROMIS Depression in Cancer questionnaires.

		Sum Sq	Mean Sq	NumDF	DenDF	F value	P-value
**PROMIS-Ca-A**	PC1	7.24	3.62	2	390	6.68	0.001 *
	PC2	0.29	0.14	2	390	0.23	0.794
**PROMIS-Ca-D**	PC1	3.42	1.71	2	370	3.15	0.044 *
	PC2	0.19	0.09	2	370	0.23	0.797
	PC3	1.16	0.58	2	370	1.30	0.274

PROMIS-Ca-A: PROMIS Anxiety in Cancer questionnaire; PROMIS-Ca-D: PROMIS Depression in Cancer questionnaire; PC: principal component; Sum Sq: sum of squares; Mean Sq: mean of squares; NumDF: numerator degrees of freedom; DenDF: denominator degrees of freedom. The * marks p-values < 0.05.

PC1 ANOVA was significant. However, posthoc testing (Table S3) did not find a significant difference between measures from cluster 1 and cluster 2 (p = 0.099) and between cluster 3 and cluster 2 measures (p = 0.093), a finding pointing out that the measures from items contaminated by the hidden, additional variable represented by PC1 (i.e. the measures from cluster 1 and cluster 3) are not significantly different from the measures from cluster 2 items, i.e. those items only affected by the variable modelled by the Rasch model).

Table S3. Posthoc analysis after ANOVA of the participants' measures from the PROMIS Anxiety and PROMIS Depression in Cancer questionnaire item clusters.

	contrast	estimate	SE	df	t.ratio	P-value
PROMIS -Ca-A	CL1 - CL2	0.12	0.07	390	1.65	0.099
	CL1 - CL3	0.27	0.07	390	3.65	0.001 *
	CL2 - CL3	0.15	0.07	390	2.00	0.093
PROMIS -Ca-D	CL1 - CL2	0.05	0.08	370	0.60	0.546
	CL1 - CL3	0.18	0.08	370	2.41	0.049 *
	CL2 - CL3	0.14	0.08	370	1.81	0.142

PROMIS-Ca-A: PROMIS Anxiety in Cancer questionnaire; PROMIS-Ca-D: PROMIS Depression in Cancer questionnaire; CL: cluster; SE: standard error; df: degrees of freedom. The * marks p-values < 0.05. Posthoc tests have been run given the ANOVA on PC1 only for both questionnaires since these two ANOVAs were the only ones beyond the significance threshold.

Regarding PC2, ANOVA did not highlight a significant difference among the measures from the three clusters of items for the PROMIS-Ca-A questionnaire (Table S2).

Regarding the PROMIS-Ca-D questionnaire, similar to what was found for the PROMIS-Ca-A, ANOVA was marginally significant for PC1 (Table S2). However, measures from cluster 1 and cluster 2 items and cluster 3 and cluster 2 items were not significantly different (Table S3; Figure S3). ANOVA found no difference for PC2 and PC3 cluster items (Table S2).

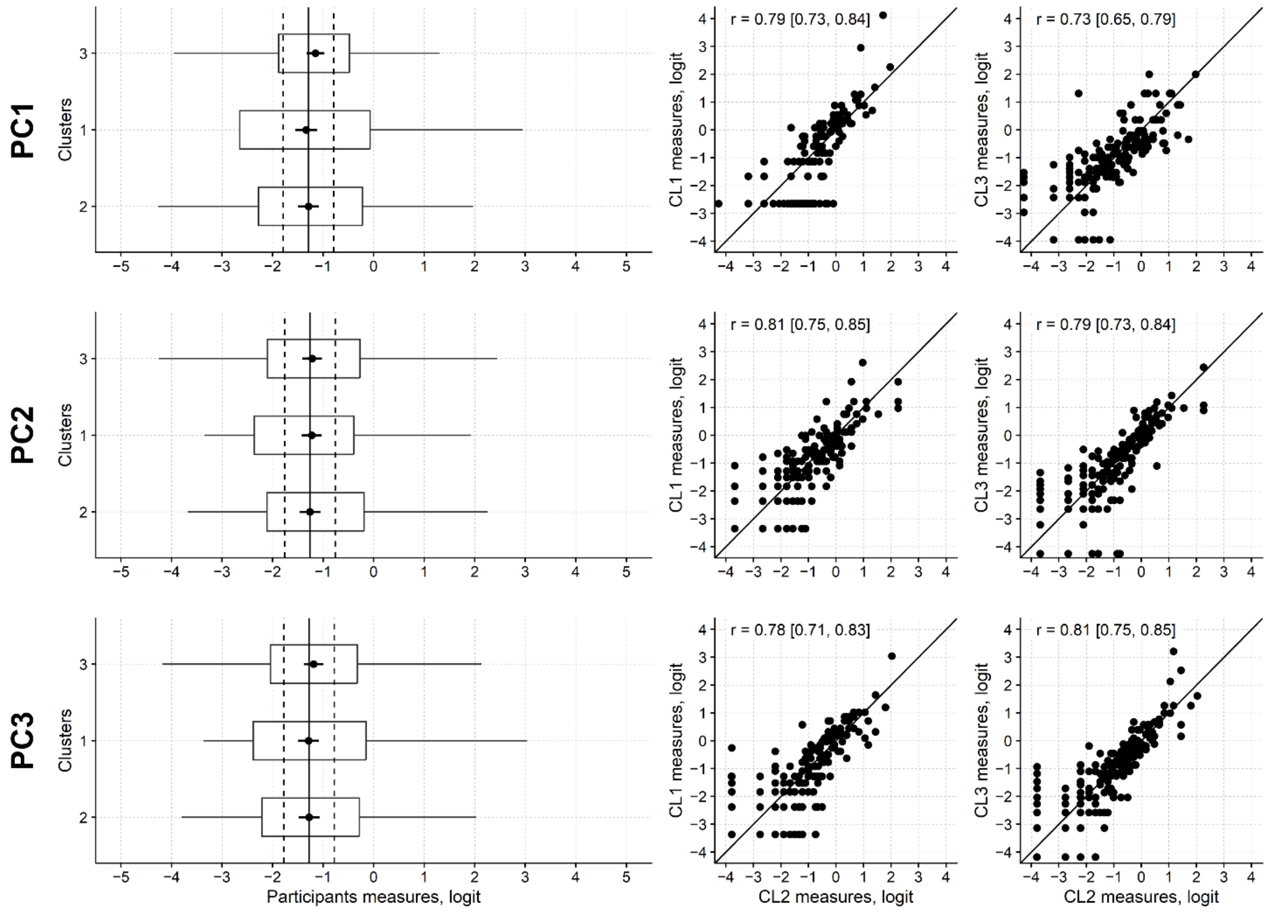

Figure S3: Dimensionality analysis of the PROMIS Depression in Cancer. Same abbreviations as Fig S2

Figures S2 and S3 also report the results of a Pearson correlation analysis to assess further the similarity between the measures from cluster 1 and cluster 2 and cluster 3 and cluster 2 items.

For both the PROMIS-Ca-A and PROMIS-Ca-D questionnaires and the different PCs the agreement between the measures from clusters 1 and 2 and 3 and 2 is reasonable, as indicated by the substantive Pearson's correlation.

For the PROMIS-Ca-A, the correlation coefficient was ≥ 0.75 (Figure S2); for the PROMIS-Ca-D, this correlation was ≥ 0.73 (Figure S3). Therefore, more than half of the variance of cluster 2 measures, i.e. those primarily reflecting the variable grasped by the Rasch model, is retained in cluster 1 and cluster 3 measures, i.e. those measures also reflecting the contaminating variables.

Considered altogether, the findings from the ANOVA and the correlation analyses point out that, even if some amount of multidimensionality affects both the PROMIS-Ca-A and the PROMIS-Ca-D questionnaire, this is too small to matter from a measurement perspective.

Appendix 2.2 – Differential Item Functioning of the PROMIS Anxiety in Cancer Questionnaire

No DIF was found for gender, education level, mental disorder, social status, single vs multiple cancer localisations, time from cancer diagnosis, and employment status (Table S4).

Item EDANX18 *"I had sudden feelings of panic"* had DIF for age (DIF contrast: -0.78 logit; p = 0.008). More precisely, the item calibration was lower (0.36 logit) in group 1 than in group 2 participants (1.15 logit). This finding means that given a middle-aged and an older person both scoring high on item EDANX18 (i.e. both complaining of feelings of sudden panic), the overall anxiety level of the older person is considerably higher than that of the middle-aged participant.

The other way round, say a middle-aged and an older person both suffer 0.7 logits of anxiety. Because of DIF, the middle-aged person will score high on item EDANX18, while the older person will score low.

Regarding the reasons for this DIF, it can be proposed that some symptoms of panic, like palpitations and shortness of breath, could be more likely attributed by old persons to a somatic impairment rather than to anxiety. The item would be affirmed as less than expected, hence the DIF.

However, given the results of a previous study [16], a single item sized about 0.75 logits is expected to be of no harm in measurements and thus can be safely ignored. Therefore, the PROMIS Anxiety in Cancer questionnaire successfully passed the DIF analysis.

Table S4. Differential Item Functioning of the PROMIS Anxiety in Cancer questionnaire.

Variable	Item	Group 1			Group 2			DIF		
		**Label**	**Calibration**	**S.E.**	**Label**	**Calibration**	**S.E.**	**Delta**	**S.E.**	**P-value**
Age	EDANX18	adults	0.36	0.12	old	1.15	0.26	0.78	0.29	0.008

In the Differential Item Functioning (DIF) analysis, items are calibrated in opposite groups of respondents. Only the variables showing sizeable (> 0.5 logits) and significant (p < 0.01) DIF are reported in this table. Variable: the variable according to which the participants' sample was split into two groups for the DIF analysis. Item: items coded according to PROMIS codes. "Label" indicates the DIF class. For age, the label "adults" indicates middle-aged participants and "old" old persons. For the BMI, "healthy" indicates a normal BMI and labels "over" and "obese" indicate overweight and obesity, respectively. Calibration: item's calibration. S.E.: standard error. Delta: absolute value of the difference between the item's calibration in Group 1 and Group 2 participants. P-value: P-value of the t-test with the null hypothesis: *"The item's calibration is not different in the two groups of participants"*.

Appendix 2.3 – Differential Item Functioning of the PROMIS Depression in Cancer Questionnaire

The same variables investigated for the DIF analysis of the PROMIS-Ca-A have also been studied for the PROMIS-Ca-D.

No DIF was found for physical activity, age, a pre-existing mental disorder, single vs multiple cancer localisations and social status. DIF was found for four variables: education level, employment status, gender and the time from diagnosis (Table S5).

Table S5. Differential Item Functioning of the PROMIS Depression in Cancer questionnaire.

Variable	Item	Group 1			Group 2			DIF		
		**Label**	**Calibration**	**S.E.**	**Label**	**Calibration**	**S.E.**	**Delta**	**S.E.**	**P-value**
Education level	EDDEP31	up to secondary	-0.36	0.13	high school	0.31	0.17	0.66	0.21	0.002
Employment status	EDDEP21	unemployed	0.28	0.12	retired	1.95	0.53	1.67	0.54	0.005
	EDDEP19	unemployed	-0.07	0.11	retired	1.11	0.38	1.17	0.40	0.006
	EDDEP12	unemployed	-1.01	0.11	retired	-1.75	0.23	0.74	0.25	0.007
	EDDEP27	unemployed	0.60	0.13	student or worker	0.05	0.14	0.55	0.19	0.006
Gender	EDDEP16	female	-0.63	0.10	male	-0.08	0.15	0.55	0.18	0.002
Time from diagnosis	EDDEP55	> 1 & ≤ 5 years	0.40	0.14	> 5 years	-0.58	0.27	0.98	0.30	0.003

Under: underweight; up to secondary: uneducated, primary or secondary school. See Table A1.1 for the remaining abbreviations.

Having a small size (< 0.75 logits), it is unlikely that DIF by gender and education level and even by time from diagnosis causes a severe malfunctioning of the measures from the questionnaire's total score [16]. However, since it is larger and affects more items, this could not be the case for the DIF by employment status.

Four items had DIF because of the employment status. The calibration of two items (EDDEP21, *"I felt that I was to blame for things",* and EDDEP19, *"I felt like I wanted to give up everything"*) was significantly larger in retired than unemployed persons. The absolute value of the DIF delta was large, 1.67 and 1.17 logit, respectively, causing an overall DIF of 2.84 logits. A third item, EDDEP12, *"I had mood swings"*, also showed DIF for retired and unemployed persons, but in this case, the calibration was higher for unemployed and retired participants. DIF of item EDDEP27 – *"I felt that no one needed me"* was more modest regarding logit.

As a control analysis, participants were measured with the set of pure items, i.e., the items free of DIF caused by employment status and the complete questionnaire. Measures from the two versions of the PROMIS-Ca-D questionnaire were compared and were found to be significantly different for none of the participants.

Even if DIF means a problem with the items' calibration, this calibration error causes no substantial artefact at the level of the single-subjects measures. Similarly to multidimensionality, even if items of the PROMIS-Ca-D were affected by DIF, this caused a negligible measurement artefact at the total score questionnaire level.

Appendix 2.4 – Score-to-measure conversion of the PROMIS Anxiety and PROMIS Depression in Cancer Questionnaires

Tables S6 and S7 provide the score-to-measure conversion for turning the total scores of the PROMIS Anxiety and PROMIS Depression in Cancer questionnaires into interval measures with the logit as the measurement unit.

Table S6. Score-to-measure conversion of the PROMIS Anxiety in Cancer questionnaire.

Score	Measure	SE	Score	Measure	SE	Score	Measure	SE
22	-4.81	1.80	52	-0.60	0.20	82	0.61	0.21
23	-3.67	0.96	53	-0.56	0.20	83	0.65	0.22
24	-3.05	0.66	54	-0.52	0.20	84	0.70	0.22
25	-2.70	0.53	55	-0.48	0.20	85	0.75	0.22
26	-2.46	0.46	56	-0.44	0.20	86	0.80	0.23
27	-2.27	0.41	57	-0.40	0.20	87	0.85	0.23
28	-2.12	0.37	58	-0.36	0.20	88	0.90	0.23
29	-1.99	0.35	59	-0.32	0.20	89	0.96	0.24
30	-1.88	0.33	60	-0.28	0.20	90	1.02	0.24
31	-1.78	0.31	61	-0.24	0.20	91	1.07	0.24
32	-1.69	0.29	62	-0.21	0.20	92	1.13	0.25
33	-1.60	0.28	63	-0.17	0.20	93	1.20	0.25
34	-1.53	0.27	64	-0.13	0.20	94	1.26	0.26
35	-1.46	0.26	65	-0.09	0.20	95	1.33	0.27
36	-1.39	0.26	66	-0.05	0.20	96	1.41	0.27
37	-1.32	0.25	67	-0.01	0.20	97	1.48	0.28
38	-1.26	0.24	68	0.03	0.20	98	1.57	0.29
39	-1.21	0.24	69	0.06	0.20	99	1.66	0.30
40	-1.15	0.23	70	0.10	0.20	100	1.75	0.32
41	-1.10	0.23	71	0.14	0.20	101	1.86	0.33
42	-1.05	0.22	72	0.18	0.20	102	1.97	0.35
43	-1.00	0.22	73	0.22	0.20	103	2.11	0.37
44	-0.95	0.22	74	0.26	0.20	104	2.26	0.40
45	-0.90	0.22	75	0.30	0.20	105	2.43	0.44
46	-0.86	0.21	76	0.35	0.20	106	2.65	0.49
47	-0.81	0.21	77	0.39	0.21	107	2.92	0.57
48	-0.77	0.21	78	0.43	0.21	108	3.31	0.70
49	-0.72	0.21	79	0.47	0.21	109	3.99	0.99
50	-0.68	0.21	80	0.52	0.21	110	5.18	1.82
51	-0.64	0.20	81	0.56	0.21	-	-	-

Score: questionnaire total score; Measure: interval measure (logit); SE: standard error of the measurement (logit; the precision of the measure estimate). The total score starts from 22 since the 22 items of the questionnaire are scored 1 to 4.

Table S7. Score-to-measure conversion of the PROMIS Depression in Cancer questionnaire.

Score	Measure	SE	Score	Measure	SE	Score	Measure	SE
30	-4.87	1.78	71	-0.59	0.17	112	0.57	0.18
31	-3.75	0.94	72	-0.57	0.17	113	0.60	0.18
32	-3.15	0.65	73	-0.54	0.17	114	0.63	0.19
33	-2.82	0.52	74	-0.51	0.17	115	0.67	0.19
34	-2.59	0.45	75	-0.48	0.17	116	0.70	0.19

35	-2.41	0.40	76	-0.46	0.17	117	0.74	0.19
36	-2.27	0.36	77	-0.43	0.16	118	0.78	0.19
37	-2.15	0.33	78	-0.40	0.16	119	0.82	0.20
38	-2.04	0.31	79	-0.37	0.16	120	0.85	0.20
39	-1.95	0.30	80	-0.35	0.16	121	0.89	0.20
40	-1.87	0.28	81	-0.32	0.16	122	0.94	0.20
41	-1.79	0.27	82	-0.29	0.16	123	0.98	0.21
42	-1.72	0.26	83	-0.27	0.16	124	1.02	0.21
43	-1.66	0.25	84	-0.24	0.16	125	1.07	0.21
44	-1.60	0.24	85	-0.21	0.16	126	1.11	0.22
45	-1.54	0.23	86	-0.19	0.16	127	1.16	0.22
46	-1.49	0.23	87	-0.16	0.16	128	1.21	0.22
47	-1.44	0.22	88	-0.13	0.16	129	1.26	0.23
48	-1.39	0.22	89	-0.11	0.16	130	1.31	0.23
49	-1.34	0.21	90	-0.08	0.16	131	1.37	0.24
50	-1.30	0.21	91	-0.05	0.16	132	1.43	0.24
51	-1.26	0.20	92	-0.03	0.16	133	1.49	0.25
52	-1.22	0.20	93	0.00	0.17	134	1.55	0.26
53	-1.18	0.20	94	0.03	0.17	135	1.62	0.26
54	-1.14	0.19	95	0.06	0.17	136	1.69	0.27
55	-1.10	0.19	96	0.08	0.17	137	1.77	0.28
56	-1.06	0.19	97	0.11	0.17	138	1.85	0.29
57	-1.03	0.19	98	0.14	0.17	139	1.94	0.30
58	-0.99	0.19	99	0.17	0.17	140	2.04	0.32
59	-0.96	0.18	100	0.20	0.17	141	2.14	0.33
60	-0.93	0.18	101	0.23	0.17	142	2.26	0.35
61	-0.89	0.18	102	0.25	0.17	143	2.39	0.38
62	-0.86	0.18	103	0.28	0.17	144	2.54	0.41
63	-0.83	0.18	104	0.31	0.17	145	2.72	0.44
64	-0.80	0.18	105	0.34	0.17	146	2.94	0.50
65	-0.77	0.17	106	0.37	0.17	147	3.23	0.57
66	-0.74	0.17	107	0.40	0.18	148	3.63	0.70
67	-0.71	0.17	108	0.44	0.18	149	4.31	1.00
68	-0.68	0.17	109	0.47	0.18	150	5.51	1.82
69	-0.65	0.17	110	0.50	0.18	-	-	-
70	-0.62	0.17	111	0.53	0.18	-	-	-

Same abbreviations as Table S6.

Appendix 2.5 – Dimensionality of the PROMIS Anxiety and PROMIS Depression in Cancer Questionnaires: Interpretive Hypotheses for the Secondary Variables

In our main dimensionality analysis (reported in Appendix 2.1), we showed that the multidimensionality affecting the two questionnaires, though present, is likely negligible in terms of the artefact it causes to the measures. Demonstrating that the multidimensionality is not practically relevant for measurement is, in essence, to affirm that the questionnaires are sufficiently unidimensional for their intended use (i.e., measuring anxiety and depression).

In this section, we will nonetheless attempt an interpretation of the primary variable highlighted by the PCA of the model residuals. We leave this additional analysis and discussion in the supplementary materials because it is an interpretation of what might be, and not a formal validity study that a latent variable deserves. Despite the limits of this exploratory analysis, it remains interesting as it allows us to focus more closely on the item content of the two questionnaires.

Below are the loadings for the PROMIS-Ca-D questionnaire items, this time considering all items with a loading > 0.40 (absolute value), rather than only those > 0.60 as in Appendix 2.1.

Table S8. Principal Component Analysis of the PROMIS Depression in Cancer– Extended loadings

**Positive loading**		**Negative loading**	
**Item**	**Loading**	**Item**	**Loading**
EDDEP41 – hopeless	0.63	EDANG09 – angry	-0.51
EDDEP26 – disappointed	0.49	EDDEP07 – away from others	-0.48
EDDEP46 – pessimistic	0.47	EDDEP16 – crying	-0.45
EDDEP48 – empty life	0.44	EDANG29 – irritable	-0.45
EDDEP39 – no reason to live	0.41	-	-

By looking at the item loadings on the first component, we can imagine that the secondary variable for the PROMIS-Ca-D is Apathy. As shown in the table, items with strong positive loading (i.e., > 0.40) are those expressing hopelessness, disappointment, pessimism, feeling life is empty, and having no reason to live. The items with strong negative loading (i.e., < -0.4) express being angry, moving away from others, feeling like crying, and feeling irritable.

Pragmatically, interpreting this component is answering the following question: what is the variable for which high levels are associated with high scores on "hopelessness" and "pessimism" items, and low scores on "anger" and "crying" items?

Since the positive-loading items describe withdrawal and immobility, while the negative-loading items describe "doing" (crying, moving away, reacting with anger), we propose this secondary variable is Apathy. In this view, a high level of Apathy leads to high scores on the first group of items and low scores on the second. It is crucial to this interpretation that Apathy is understood as a construct independent of Depression. This condition is essential because the principal component is, by definition, orthogonal (i.e., uncorrelated) to the main variable captured by the Rasch model.

The following table reports the items with absolute loading ≥ 0.4 for the PROMIS-Ca-A questionnaire.

Table S9. Principal Component Analysis of the PROMIS Depression in Anxiety – Extended loadings

**Positive loading**		**Negative loading**	
**Item**	**Loading**	**Item**	**Loading**
EDANX02 – frightened	0.65	EDANX51 – difficult to relax	-0.43

EDANX01 – fearful	0.50	EDANX26 – fidgety	-0.43
EDANX03 – scared me when nervous	0.46	EDANX46 – nervous	-0.42

Following the same reasoning, looking at the item content, we can hypothesize that the secondary variable indicated by the first PC of the model residuals is psychological insight (or awareness) of one's anxiety disorder, which contrasts with somatization.

A person with high awareness of their disorder may be more "frightened" when the anxiety presents. Conversely, a person with poor awareness may tend more easily toward somatization, reporting difficulty relaxing and restlessness, rather than the emotion of fear. In the first patient, the one with high awareness, these somatic symptoms might be kept under control precisely because of this high level of insight. This suppression of the physical disturbances, however, leaves space for the "fear" to emerge.

It is important to emphasize that these are only sensible, plausible hypotheses. Whether these variables truly exist and what they represent remains to be discovered.

Furthermore, these interpretations do not preclude others. For example, in the case of the PROMIS-Ca-A, the secondary variable could also represent the temporal presentation of the anxiety. People who suffer from acute poussées (attacks) of anxiety will more likely suffer intense, violent disturbances that lead to fear (e.g., panic). Conversely, people suffering from generalized anxiety live in a continuous context of anxiety that pervasively leads them to be restless and have difficulty relaxing.

Appendix 3 – Amending for the disordered thresholds of the PROMIS Anxiety and PROMIS Depression in cancer questionnaires

As reported in the main text, both the PROMIS Anxiety and the PROMIS Depression in Cancer questionnaires showed disordered modal thresholds. For both questionnaires, category 2 was never modal (i.e. it was never the most likely) along the latent variable line. Despite this, both questionnaires presented ordered average categories' measures, and precisely because of this last finding, the primary analysis continued without acting on the items categories' structure.

Because of the ongoing discussion in the Rasch community about the need for ordered modal thresholds in addition to ordered average categories' measures to conclude the proper functioning of categories (Appendix 1 – Supplementary Materials 3), in these supplementary analyses, the Rasch analysis of the PROMIS-Ca-Anxiety and PROMIS-Ca-Depression is repeated after amending the disordered modal thresholds.

Appendix 3.1 – PROMIS Anxiety in Cancer questionnaire

In this analysis, the original categories 2 ("Rarely") and 3 ("Sometimes") were combined into a new category 2 ("Rarely or Sometimes"). The original categories 4 ("Often") and 5 ("Always") were shifted one numeral downward and labelled 3 and 4, respectively. Category 1 remained "Never" (Table S8).

Table S8: labelling of the revised categories of the PROMIS Anxiety in Cancer Questionnaire

Original		Revised	
**Numeral**	**Description**	**Numeral**	**Description**
1	Never	1	Never
2	Rarely	2	Rarely or Sometimes
3	Sometimes		
4	Often	3	Often
5	Always	4	Always

After this rearrangement, categories and modal thresholds were ordered (Table S9).

Table S9: PROMIS Anxiety in Cancer Questionnaire – revised categories

Label	Count	Observed average	Modal thresholds
1	2352	-2.73	-
2	1546	-0.92	-1.57
3	443	-0.03	0.76
4	345	0.78	0.81

The PROMIS Anxiety in Cancer Questionnaire items are scored in five categories: 1 to 5. Because of disordered modal (Andrich's) thresholds, the item categories were recoded by collapsing the original categories 2 and 3. The revised items have thus four categories, 1 to 4. Observed average: average measure of the participants scoring in a category (the average measure of all the participants scoring 1 in any item was -2.73 logits, that of the participants scoring 2 in any item was -0.92 logits and so on). If, and only if, there is a monotonic relationship between the categories numerals and the measured quantity, the participant mean measure of those participants choosing a higher category is higher than that of those participants choosing a lower one.

Figure S4A shows the Bland-Altman plot of the agreement between the calibrations of the PROMIS-Ca-A items with the original categories structure (categories 1 to 5, ordered average categories measures and disordered modal thresholds) and the revised categories (categories 1 to 4, ordered average categories measures and modal thresholds). Threshold disordering has a minor impact on item estimates, as shown by a maximum difference between calibration estimates of 0.25 logits in absolute value. The fit of the items to the model (Figure S4B) is also substantially unaffected by threshold disordering.

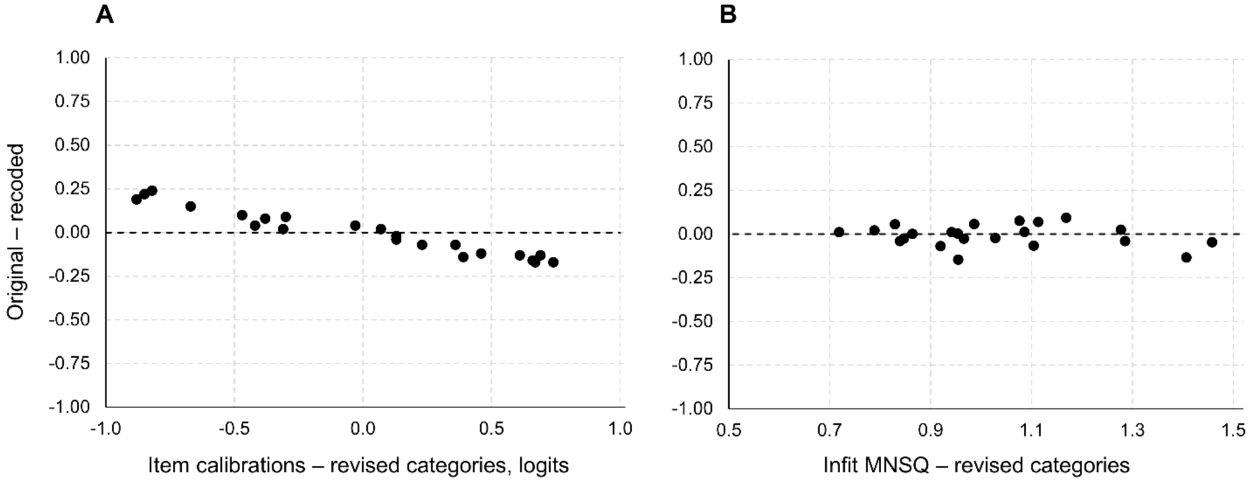

Figure S4: PROMIS Anxiety in Cancer questionnaire – revised vs original categories. These Bland-Altman plots contrast the item calibration (A) and the fit of the items to the model (B) of the PROMIS-Ca-A questionnaire with the original and the revised categories' structure. Model fit is quantified with the infit mean square (MNSQ). The PROMIS-Ca-A items are scored 1 to 5. In the revised questionnaire, the original categories 2 and 3 have been collapsed into a novel category 2, and the item's categories are relabelled 1 to 4 to amend disordered modal thresholds. Since the revised questionnaire has ordered categories average measures, and modal thresholds, this questionnaire is used as the criterion in this supplementary analysis. Original – recoded (y-axis): in plot A, difference between the items calibration of the questionnaire with the original 5-categories structure minus the items calibration of the revised one. In plot B, original – recoded reports the difference between the infit MNSQ values.

Finally, for completeness, the score-to-measure conversion of the PROMIS-Ca-Anxiety questionnaire with the revised categories structure is provided in Table S10 for those scholars requiring ordered average categories measures and modal thresholds to turn questionnaire scores into measures.

Table S10: score to measure conversion of the PROMIS Anxiety in Cancer Questionnaire with revised categories' labels

Score	Measure	S.E.	Score	Measure	S.E.
22	-5.99	1.84	56	0.24	0.26
23	-4.75	1.02	57	0.30	0.26
24	-4.01	0.74	58	0.37	0.26
25	-3.56	0.62	59	0.44	0.26
26	-3.22	0.55	60	0.50	0.26
27	-2.95	0.50	61	0.57	0.26
28	-2.72	0.47	62	0.63	0.26
29	-2.51	0.44	63	0.70	0.26
30	-2.33	0.42	64	0.76	0.26
31	-2.16	0.40	65	0.83	0.26
32	-2.00	0.39	66	0.90	0.26

33	-1.86	0.38	67	0.97	0.26
34	-1.72	0.36	68	1.04	0.26
35	-1.59	0.35	69	1.11	0.27
36	-1.47	0.35	70	1.18	0.27
37	-1.35	0.34	71	1.25	0.27
38	-1.24	0.33	72	1.33	0.28
39	-1.14	0.32	73	1.41	0.28
40	-1.03	0.32	74	1.49	0.29
41	-0.94	0.31	75	1.57	0.30
42	-0.84	0.30	76	1.66	0.30
43	-0.75	0.30	77	1.76	0.31
44	-0.66	0.29	78	1.86	0.32
45	-0.58	0.29	79	1.97	0.34
46	-0.49	0.29	80	2.09	0.35
47	-0.41	0.28	81	2.22	0.38
48	-0.33	0.28	82	2.37	0.40
49	-0.26	0.27	83	2.54	0.44
50	-0.18	0.27	84	2.76	0.49
51	-0.11	0.27	85	3.03	0.56
52	-0.04	0.27	86	3.41	0.69
53	0.03	0.26	87	4.07	0.98
54	0.10	0.26	88	5.25	1.81
55	0.17	0.26	-	-	-

Appendix 3.2 – PROMIS Depression in Cancer questionnaire

Like the PROMIS-Ca-Anxiety questionnaire, the Depression questionnaire also showed disordered modal thresholds, with the threshold between categories 1 and 2 with a higher calibration of that between categories 2 and 3.

Similar to the anxiety questionnaire, the analysis was repeated after merging the original categories 2, “Rarely” and 3 “Sometimes” in the new category 2 “Rarely or Sometimes”, a solution that allowed for ordered average measures and thresholds to be obtained (Table S11).

Table S11: PROMIS Depression in Cancer Questionnaire – revised categories

Label	Count	Observed average	Modal thresholds
1	3718	-2.98	-
2	1680	-0.95	-1.43
3	588	0.03	0.56
4	404	0.74	0.87

Same abbreviations as in Table S9.

Figure S5 shows the Bland-Altman plots of the agreement between the item calibration and the fit to the model with the revised and the original categories. Similarly to the PROMIS-Ca-A, threshold disordering had only minor consequences on item estimates.

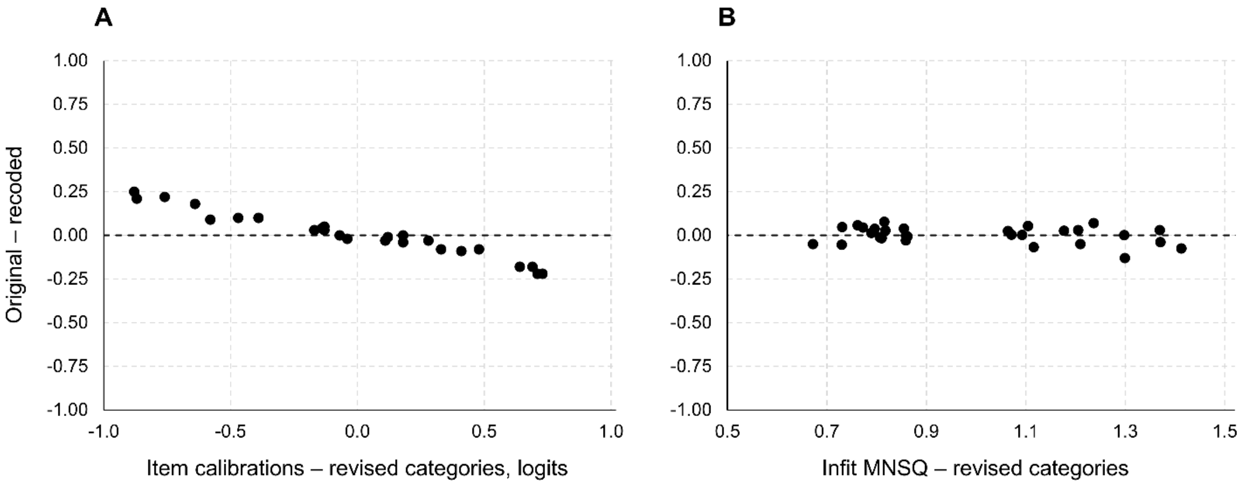

Figure S5: PROMIS Depression in Cancer questionnaire – revised vs original categories. Same legend as Figure S4.

The score to measure conversion of the PROMIS-Ca-Depression questionnaire is provided next (Table S12).

Table S12: score-to-measure conversion of the PROMIS Depression in Cancer Questionnaire with revised categories

Score	Measure	S.E.	Score	Measure	S.E.
30	-6.21	1.84	76	0.17	0.22
31	-4.98	1.02	77	0.22	0.22
32	-4.26	0.73	78	0.27	0.22
33	-3.82	0.61	79	0.32	0.22
34	-3.49	0.53	80	0.36	0.22
35	-3.24	0.48	81	0.41	0.22
36	-3.02	0.45	82	0.46	0.22
37	-2.83	0.42	83	0.51	0.22
38	-2.67	0.40	84	0.56	0.22
39	-2.52	0.38	85	0.61	0.22
40	-2.38	0.36	86	0.66	0.22
41	-2.25	0.35	87	0.71	0.22
42	-2.13	0.34	88	0.76	0.22
43	-2.02	0.33	89	0.81	0.22
44	-1.92	0.32	90	0.86	0.23
45	-1.82	0.31	91	0.91	0.23
46	-1.72	0.31	92	0.96	0.23
47	-1.63	0.30	93	1.01	0.23
48	-1.54	0.29	94	1.07	0.23
49	-1.46	0.29	95	1.12	0.23

50	-1.38	0.28	96	1.18	0.24
51	-1.30	0.28	97	1.23	0.24
52	-1.22	0.27	98	1.29	0.24
53	-1.15	0.27	99	1.35	0.25
54	-1.08	0.26	100	1.41	0.25
55	-1.01	0.26	101	1.47	0.25
56	-0.94	0.26	102	1.54	0.26
57	-0.88	0.25	103	1.61	0.26
58	-0.81	0.25	104	1.68	0.27
59	-0.75	0.25	105	1.75	0.27
60	-0.69	0.25	106	1.83	0.28
61	-0.63	0.24	107	1.91	0.29
62	-0.57	0.24	108	2.00	0.30
63	-0.51	0.24	109	2.09	0.31
64	-0.46	0.24	110	2.19	0.32
65	-0.40	0.24	111	2.30	0.34
66	-0.35	0.23	112	2.42	0.36
67	-0.29	0.23	113	2.55	0.38
68	-0.24	0.23	114	2.71	0.41
69	-0.19	0.23	115	2.89	0.44
70	-0.13	0.23	116	3.11	0.49
71	-0.08	0.23	117	3.39	0.57
72	-0.03	0.23	118	3.78	0.70
73	0.02	0.22	119	4.46	0.99
74	0.07	0.22	120	5.66	1.82
75	0.12	0.22	-	-	-

References

1. Adams, R.J.; Wu, M.L.; Wilson, M. The Rasch Rating Model and the Disordered Threshold Controversy. *Educational and Psychological Measurement* **2012**, *72*, 547–573, doi:10.1177/0013164411432166.
2. Andrich, D. An Expanded Derivation of the Threshold Structure of the Polytomous Rasch Model That Dispels Any “Threshold Disorder Controversy.” *Educational and Psychological Measurement* **2013**, *73*, 78–124, doi:10.1177/0013164412450877.
3. Linacre, J.M. *Winsteps® Rasch Measurement Computer Program User's Guide.*; Version 5.6.0.; Winsteps.com: Portland, Oregon, 2023;
4. García-Pérez, M.A. An Analysis of (Dis)Ordered Categories, Thresholds, and Crossings in Difference and Divide-by-Total IRT Models for Ordered Responses. *Span J Psychol* **2017**, *20*, E10, doi:10.1017/sjp.2017.11.
5. Linacre, J. Category Disordering (Disordered Categories) vs. Threshold Disordering (Disordered Thresholds). *Rasch Measurement Transactions* **1999**, *13*, 675.
6. Wright, B.; Masters, G.N. Computation of OUTFIT and INFIT Statistics. *Rasch Measurement Transactions* **1990**, *3*, 84–85.
7. Cohen, J. *Statistical Power Analysis for the Behavioral Sciences*; 2nd ed.; Routledge: New York, 1988; ISBN 978-0-203-77158-7.
8. Swinscow, T.D. Statistics at Square One. XV--The Chi-Squared Tests (Continued). *Br Med J* **1976**, *2*, 513–514, doi:10.1136/bmj.2.6034.513.
9. Schulz, M. The Standardization of Mean-Squares: Wilson-Hilferty. *Rasch Measurement Transactions* *16*, 879.
10. Zaina, F.; Ferrario, I.; Caronni, A.; Scarano, S.; Donzelli, S.; Negrini, S. Measuring Quality of Life in Adults with Scoliosis: A Cross-Sectional Study Comparing SRS-22 and ISYQOL Questionnaires. *J Clin Med* **2023**, *12*, 5071, doi:10.3390/jcm12155071.
11. Linacre, J.; Wright, B. Chi-Square Fit Statistics. *Rasch Measurement Transactions* **1994**, *8*, 350.
12. Baghaei, P. The Rasch Model as a Construct Validation Tool. *Rasch Measurement Transactions* **2008**, *22*, 1145–1146.
13. Linacre, J.M. DIF - DPF - Bias - Interactions Concepts Available online: https://www.winsteps.com/winman/difconcepts.htm.
14. Tennant, A.; Pallant, J. DIF Matters: A Practical Approach to Test If Differential Item Functioning Makes a Difference. *Rasch measurement transactions* **2007**, *20*, 1082–1084.
15. Smith Jr, E.V. Detecting and Evaluating the Impact of Multidimensionality Using Item Fit Statistics and Principal Component Analysis of Residuals. *Journal of applied measurement* **2002**, *3*, 205–231.
16. Caronni, A.; Scarano, S. Generalisability of the Barthel Index and the Functional Independence Measure: Robustness of Disability Measures to Differential Item Functioning. *Disabil Rehabil* **2024**, 1–12, doi:10.1080/09638288.2024.2391554.
17. Fisher, W.P. Reliability, Separation, Strata Statistics. *Rasch measurement transactions* **1992**, *6*, 238.
18. Wright, B. Reliability and Separation. *Rasch measurement transactions* **1996**, *9:4*, 472.
19. Wright, B.D. Separation, Reliability and Skewed Distributions: Statistically Different Levels of Performance. *Rasch Meas Trans* **2001**, *14*, 786.
20. Tennant, A.; Conaghan, P.G. The Rasch Measurement Model in Rheumatology: What Is It and Why Use It? When Should It Be Applied, and What Should One Look for in a Rasch Paper? *Arthritis Care & Research* **2007**, *57*, 1358–1362, doi:10.1002/art.23108.
21. Linacre, J.M. Sample Size and Item Calibration Stability. *Rasch Measurement Transactions* **1994**, *7*, 328.
